# Duplication and Functional Divergence of Branched-Chain Amino Acid Biosynthesis Genes in Aspergillus nidulans

**DOI:** 10.1128/mBio.00768-21

**Published:** 2021-06-22

**Authors:** Joel T. Steyer, Damien J. Downes, Cameron C. Hunter, Pierre A. Migeon, Richard B. Todd

**Affiliations:** a Department of Plant Pathology, Kansas State University, Manhattan, Kansas, USA; Universidade de Sao Paulo

**Keywords:** LEU3, amino acid biosynthesis, branched-chain amino acid metabolism, filamentous fungi, gene regulation, isopropylmalate, leucine, primary metabolism, transcription factors, valine

## Abstract

Fungi, bacteria, and plants, but not animals, synthesize the branched-chain amino acids: leucine, isoleucine, and valine. While branched-chain amino acid (BCAA) biosynthesis has been well characterized in the yeast Saccharomyces cerevisiae, it is incompletely understood in filamentous fungi. The three BCAAs share several early biosynthesis steps before divergence into specific pathways. In Aspergillus nidulans, the genes for the first two dedicated steps in leucine biosynthesis have been characterized, but the final two have not. We used sequence searches of the A. nidulans genome to identify two genes encoding β-isopropylmalate dehydrogenase, which catalyzes the penultimate step of leucine biosynthesis, and six genes encoding BCAA aminotransferase, which catalyzes the final step in biosynthesis of all three BCAA. We have used combinations of gene knockouts to determine the relative contribution of each of these genes to BCAA biosynthesis. While both β-isopropylmalate dehydrogenase genes act in leucine biosynthesis, the two most highly expressed BCAA aminotransferases are responsible for BCAA biosynthesis. We have also characterized the expression of leucine biosynthesis genes using reverse transcriptase-quantitative PCR and found regulation in response to leucine availability is mediated through the Zn(II)_2_Cys_6_ transcription factor LeuB.

## INTRODUCTION

The branched-chain amino acids (BCAA) leucine, isoleucine, and valine are essential dietary amino acids in mammals. Leucine levels provide an acute signal for nutrient availability to control the protein kinase mTORC1 (mammalian Target of Rapamycin Complex 1), which is a pleiotropic regulator of many cellular processes, including cell growth, protein biosynthesis, the response to nutrient availability, and autophagy (1, 2). Unlike mammals, fungi synthesize BCAA for use in protein biosynthesis and as precursors for secondary metabolites (3). BCAA biosynthesis genes also play important roles during infection for fungal pathogens. BCAA auxotrophs in the opportunistic human fungal pathogens Cryptococcus neoformans, Candida albicans, and Aspergillus fumigatus show decreased pathogenicity (4–9), and the plant pathogens Magnaporthe oryzae and Fusarium graminearum require BCAA biosynthesis genes for full virulence (10–15). Therefore, the enzymes for BCAA biosynthesis are potential drug and antifungal agent targets.

Synthesis of the three BCAAs occurs via a dichotomous biochemical pathway and is well characterized in Saccharomyces cerevisiae (16). Studies of BCAA biosynthesis in A. fumigatus, Aspergillus niger, and Aspergillus nidulans have revealed both divergence from and similarity to S. cerevisiae (5, 17–20). For example, both A. nidulans and S. cerevisiae encode a single α-isopropylmalate isomerase (19, 21). A. fumigatus encodes two functional dihydroxyacid dehydratases (5), whereas S. cerevisiae encodes only one (22), whereas the production of α-isopropylmalate (α-IPM) in S. cerevisiae is carried out by two α-IPM synthetases, encoded by *LEU4* and *LEU9* (23–26), but only a single gene in A. nidulans, *leuC*, encodes a functional *LEU4*/*LEU9* ortholog (27). The final two steps in leucine biosynthesis are catalyzed sequentially by β-isopropylmalate (β-IPM) dehydrogenase and the bidirectional BCAA aminotransferase (BAT), which also produces isoleucine and valine and catalyzes the first step in BCAA catabolism (28). Although the genes encoding these enzymes have been characterized in S. cerevisiae, their A. nidulans orthologs are unknown.

Leucine biosynthesis in A. nidulans is thought to be regulated by the Zn(II)_2_Cys_6_ transcription factor LeuB (19). LeuB regulates target genes through either consensus CCGN_4_CGG DNA-binding sites, like its S. cerevisiae counterpart Leu3p, or a nonconsensus CCGN_5_CGG motif, which is also the target of TamA (27, 29). Regulation by LeuB and Leu3p is controlled by feedback inhibition through intracellular levels of free leucine (19, 27, 30). When leucine is abundant, it interacts with the α-IPM synthetase Leu4p to inhibit its activity and decrease production of the leucine biosynthesis pathway intermediate α-IPM (31). Leu3p acts as a repressor when α-IPM levels are low but is converted to an activator by binding of α-IPM (32). A. nidulans leucine biosynthesis loss-of-function mutants *luA1* (affecting α-IPM isomerase) and *leuC*Δ (affecting α-IPM synthetase), which are predicted to have increased or decreased α-IPM levels, respectively, show that LeuB responds similarly to Leu3p (27). This mechanism is conserved in A. fumigatus LeuB, which regulates leucine biosynthesis and iron acquisition genes (9, 33).

In addition to regulating leucine biosynthesis genes, Leu3p and LeuB regulate expression of their respective NADP-dependent glutamate dehydrogenase (NADP-GDH)-encoding genes, *GDH1* and *gdhA* (9, 19, 33, 34). Consistent with feedback inhibition of leucine biosynthesis through LeuB, exogenous leucine also negatively affects A. nidulans *gdhA*-*lacZ* reporter gene expression (27). NADP-GDH assimilates nitrogen nutrients producing glutamate, which is the amino donor in the final step of leucine biosynthesis. Coregulation of NADP-GDH production by the leucine pathway transcription factor is thought to ensure glutamate levels sufficient to sustain leucine production (16). It has been suggested that, through the feedback mechanisms provided by leucine levels and the coregulation of NADP-GDH expression, leucine, which is one of the most common protein-incorporated amino acids and one of the least abundant free cellular amino acids, acts as a general sensor for amino acid abundance (16).

The A. nidulans leucine biosynthesis pathway genes encoding α-IPM synthase (*leuC*) and α-IPM isomerase (*luA*) have been characterized previously (19, 27). In this study, we characterize the two genes encoding β-IPM dehydrogenases and six genes predicted to encode branched-chain amino acid aminotransferases, which together constitute the final two steps of the leucine biosynthesis pathway in A. nidulans. We demonstrate roles for both β-IPM dehydrogenase genes and reveal that only two of the six branched-chain amino acid aminotransferases are major contributors to BCAA production. We have also investigated the regulation of these genes by LeuB and leucine.

## RESULTS

### Identification of the two A. nidulans β-isopropylmalate dehydrogenase genes.

**FIG 1:**
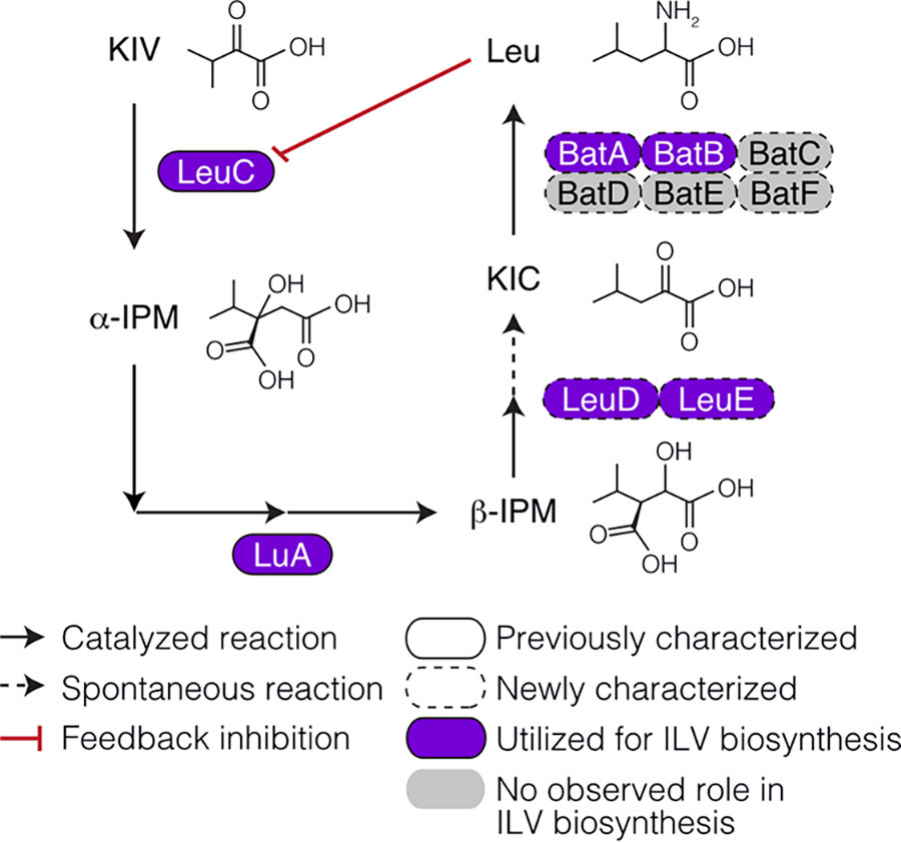
Leucine biosynthesis in Aspergillus nidulans. Pathway of committed leucine (Leu) biosynthesis enzymes (rounded rectangles). The stages involving generation of α-isopropylmalate (α-IPM) from α-ketoisovalerate (KIV) by α-IPM synthetase (LeuC) and subsequent conversion to β-isopropylmalate (β-IPM) by α-IPM isomerase (LuA) have previously been characterized. The two β-IPM dehydrogenase enzymes (LeuD and LeuE), which generate α-ketoisocaproate (KIC), and two BCAA aminotransferases (BatA and BatB), which also function in isoleucine and valine biosynthesis and isoleucine, leucine, and valine (ILV) catabolism, were characterized in this work from eight candidate coding genes.

The penultimate step in leucine biosynthesis is catalyzed by β-IPM dehydrogenase (Fig. 1). A single gene in yeast, *LEU2*, encodes β-IPM dehydrogenase (35, 36), whereas in A. niger, two enzymes, Leu2A and Leu2B, encoded by separate genes, carry out this role (18). Two A. nidulans β-IPM dehydrogenase enzymes, encoded by AN0912 and AN2793, were identified in BLASTp searches with S. cerevisiae Leu2p as the query. AN0912 and AN2793 showed high levels of similarity and identity with Leu2p, Leu2A, and Leu2B, with AN0912 most similar to Leu2A and AN2793 most similar to Leu2B (Table 1). AN0912 and AN2793 showed 50.5% identity and 67.3% similarity with each other. Alignment of the five proteins revealed strong conservation throughout the protein, including in the substrate binding loop and NAD binding motif (see Fig. S1 in the supplemental material). We designated AN0912 *leuD* and AN2793 *leuE. leuD* is found on chromosome VIII in a region of highly conserved gene colinearity in all 27 Aspergillus species genomes available at FungiDB (Fig. S2A). In contrast, *leuE* is located on chromosome VI and lacks colinearity with its 24 orthologs in the 27 Aspergillus species (Fig. S2B).

**TABLE 1:** Pairwise protein sequence comparisons of β-IPM dehydrogenases

Protein^*a*^	Systematic name	Leu2p		Leu2A		Leu2B	
		% Identity	% Similarity	% Identity	% Similarity	% Identity	% Similarity
Leu2p	YCL018W	100	100	ND^*b*^	ND	ND	ND
LeuD	AN0912	62.8	79.6	87.7	94.5	50.7	66.3
LeuE	AN2793	50.1	64.8	53.3	67.7	84.9	92.2

a A. nidulans LeuD and LeuE β-IPM dehydrogenase full-length protein sequences were aligned pairwise and compared with S. cerevisiae Leu2p and A. niger Leu2A and Leu2B.

b ND, not determined.

10.1128/mBio.00768-21.1 FIG S1 Clustal Omega alignment of β-isopropylmalate dehydrogenases. Clustal Omega alignment of Leu2p from S. cerevisiae (S. cer) with AN0912 (LeuD) and AN2793 (LeuE) from A. nidulans (A. nid) and Leu2A and Leu2B from A. niger (A. nig). The substrate-binding loop (green) and the NAD-binding motif (blue) are boxed. Shading was performed with Boxshade with a minimum of 0.6 identity (black) or similarity (gray). Download FIG S1, PDF file, 0.6 MB. Copyright © 2021 Steyer et al. 2021 Steyer et al. https://creativecommons.org/licenses/by/4.0/ This content is distributed under the terms of the Creative Commons Attribution 4.0 International license.

10.1128/mBio.00768-21.2 FIG S2 Colinearity of β-isopropylmalate dehydrogenase genes in aspergilli. The colinearity of syntenic regions for (A) AN0912 (*leuD*) and (B) AN2793 (*leuE*) was illustrated using the GBrowse genome browser of FungiDB with genomes displayed in the following order: Aspergillus nidulans FGSC A4, Aspergillus *ochraceoroseus* IBT 24754, Aspergillus sydowii CBS 593.65, Aspergillus versicolor CBS 583.65, Aspergillus *aculeatus* ATCC 16872, Aspergillus *carbonarius* ITEM 5010, Aspergillus *brasiliensis* CBS 101740, Aspergillus tubingensis CBS 134.48, Aspergillus *kawachii* IFO 4308, Aspergillus *luchuensis* CBS 106.47, Aspergillus niger ATCC 1015, Aspergillus niger ATCC 13496, Aspergillus niger CBS 513.88, Aspergillus niger strain N402 (ATCC 64974), Aspergillus oryzae RIB40, Aspergillus flavus NRRL3357, Aspergillus *steynii* IBT 23096, Aspergillus terreus NIH2624, Aspergillus *campestris* IBT 28561, Aspergillus fumigatus A1163, Aspergillus fumigatus Af293, Aspergillus fischeri NRRL 181, Aspergillus *novofumigatus* IBT 16806, Aspergillus clavatus NRRL 1, Aspergillus *glaucus* CBS 516.65, Aspergillus *wentii* DTO 134E9, Aspergillus *zonatus* CBS 506.65. Download FIG S2, PDF file, 2 MB. Copyright © 2021 Steyer et al. 2021 Steyer et al. https://creativecommons.org/licenses/by/4.0/ This content is distributed under the terms of the Creative Commons Attribution 4.0 International license.

**FIG 2:**
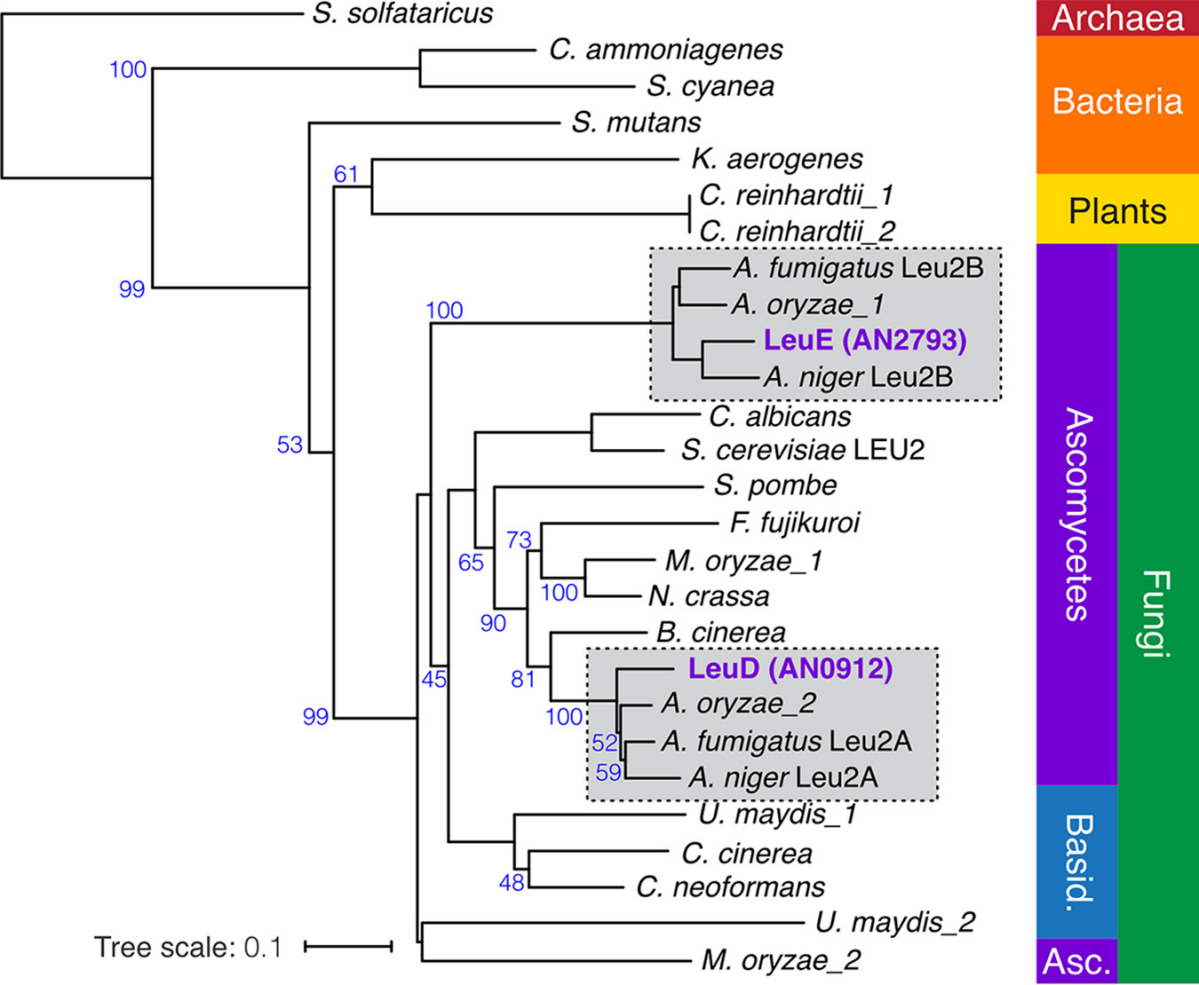
Phylogenetic analysis of β-IPM dehydrogenases. Unrooted phylogeny of β-IPM dehydrogenases is shown. Bootstrap support (100 replicates) greater than 40% is shown. Protein sequences for A. nidulans were downloaded from AspGD, sequences for S. cerevisiae were downloaded from SGD, and all other sequences came from Pfam or NCBI. *Archaea*, Sulfolobus solfataricus (Q9UXB2.1); *Bacteria*, Corynebacterium ammoniagenes (D5NZR1.1), Klebsiella aerogenes (WP_077203698.1), Streptococcus mutans (Q8DTG3.1), Saccharomonospora cyanea (H5XNC6.1); Basidiomycota (Basid.), Coprinopsis cinerea (A8NYJ8.1), Cryptococcus neoformans (Q5KP37.1), Ustilago maydis (1, XP_011387179.1; 2, XP_011391948.1); Ascomycota (Asc.), A. fumigatus Leu2A (Q4WRM6.1), Leu2B (Q4WLG7.1), A. nidulans LeuD (AN0912), LeuE (AN2793), A. niger Leu2A (P87256.1), Leu2B (P87257.1), A. oryzae (1, Q2TYA5.1; 2, Q877A9.1), *Botrytis cinerea* (XP_001546815.1) Candida albicans (C4YTB1.1), Fusarium *fujikuroi* (C1L3C2.1), *M. oryzae* (1, G4N5B0.1; 2, G4NIK0.1), Neurospora crassa (P34738.2), Saccharomyces cerevisiae Leu2p (YCL018W), Schizosaccharomyces pombe (P18869.1); *Planta*, Chlamydomonas reinhardtii (1, A8I7N4.1; 2, A8I7N8.1). The scale bar corresponds to the branch length for an expected number of 0.1 substitutions per site. The two distinct Aspergillus clades are boxed.

We investigated the relationships of the two A. nidulans β-IPM dehydrogenase genes through construction of a phylogenetic tree (Fig. 2). LeuD and LeuE formed distinct clades with their respective Aspergillus orthologs. The LeuD clade is consistent with the position of A. nidulans in the fungal evolutionary tree (37), whereas the LeuE clade lies between the Ascomycota and the Basidiomycota clades.

### *leuD* and *leuE* both function in leucine biosynthesis.

**FIG 3:**
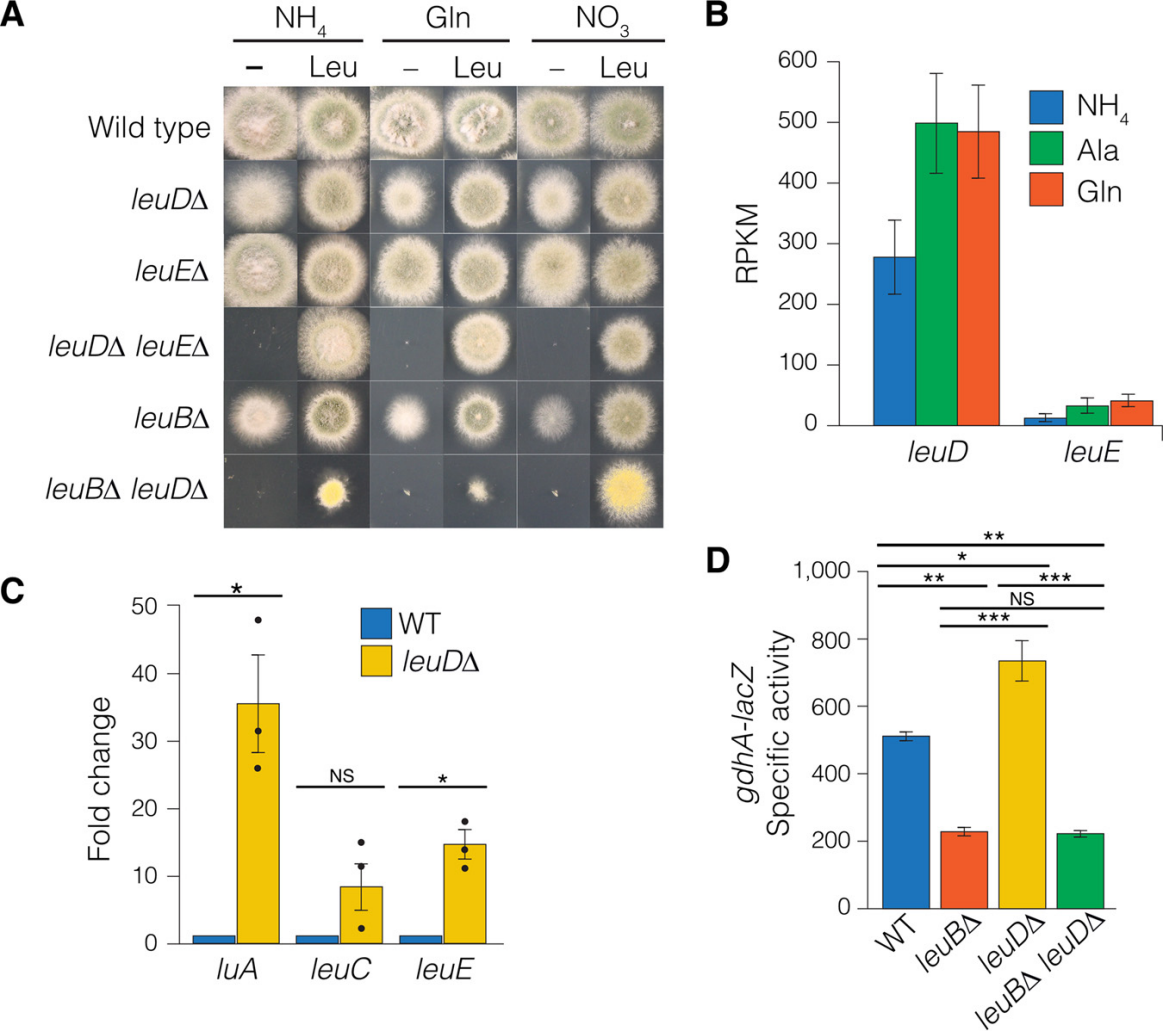
*leuD* encodes the major β-IPM dehydrogenase. (A) Wild-type (MH1), *leuD*Δ (RT411), *leuE*Δ (RT413), *leuD*Δ *leuE*Δ (RT444), *leuBΔ* (RT452), and *leuBΔ leuDΔ* (RT460) strains were grown at 37°C for 2 days on solid supplemented ANM with or without 2 mM leucine (Leu) and with 10 mM ammonium (NH_4_), glutamine (Gln), and nitrate (NO_3_) as the nitrogen source. Note that the yellow colony color of RT460 is due to the *yA1* conidial color mutation and is unrelated to the *leuB*Δ *leuDΔ* phenotype. (B) Mean reads per kilobase per million mapped reads (RPKM) from RNA-seq of MH1 grown at 37°C for 16 h in supplemented liquid ANM with 10 mM ammonium (NH_4_), glutamine (Gln), and alanine (Ala). (C) RT-qPCR quantification of mean fold change in transcript expression in *leuDΔ* (RT411) strain compared to the wild type (MH1) grown at 37°C for 16 h in supplemented liquid ANM–10 mM ammonium and 2 mM leucine. Bars indicate the mean fold change from three independent biological replicates (circles). *, *P* ≤ 0.05. NS, not significant using two-tailed Student's *t* test with equal distribution. (D) LacZ specific activity for wild-type (MH12101), *leuBΔ* (MH12181), *leuD*Δ (RT458), and *leuBΔ leuD*Δ (RT460) strains, which contain the −753 bp *gdhA*-*lacZ* reporter construct. Strains were grown at 37°C for 16 h in supplemented liquid ANM with 10 mM ammonium and 2 mM leucine (*n* = 3). *, *P* ≤ 0.05; **, *P* ≤ 0.001; ***, *P* ≤ 0.0001; NS, not significant; using one-way ANOVA. For panels B to D, error bars depict standard error of the mean (*N* = 3).

To determine whether *leuD* and *leuE* are functional genes, we generated deletion mutants by gene replacement (Fig. S3A; see Materials and Methods). Deletion of genes required for leucine biosynthesis results in leucine auxotrophy (19, 27), yet neither *leuDΔ* nor *leuE*Δ strain conferred strict leucine auxotrophy (Fig. 3A). However, while the *leuEΔ* strain grew similarly to the wild type in the absence of leucine, the *leuDΔ* mutant showed reduced growth compared with the wild type unless supplemented with exogenous leucine. Transformation of the *leuD* gene into the *leuD*Δ mutant restored leucine prototrophy (Fig. S4A). To determine whether the leaky nature of the *leuD*Δ leucine auxotrophy resulted from LeuE activity, we constructed a *leuD*Δ *leuE*Δ double mutant by meiotic crossing and found that the double mutant was a strict auxotroph, showing growth only when supplemented with exogenous leucine (Fig. 3A). Leucine supplementation of a C. neoformans auxotroph lacking α-IPM isomerase is possible when glutamine or asparagine, but not ammonium, is the nitrogen source (6). In contrast, the *leuC*Δ mutant lacking α-IPM isomerase can be supplemented on ammonium (27). Likewise, the *leuDΔ leuEΔ* leucine auxotrophy could be supplemented on the preferred nitrogen sources ammonium and glutamine and on the alternative nitrogen source nitrate (Fig. 3A). Therefore, regulation of leucine uptake in A. nidulans is not regulated by nitrogen metabolite repression.

10.1128/mBio.00768-21.3 FIG S3 Gene knockout cassettes. Strategy for the deletion of two candidate β-IPM dehydrogenase-encoding genes, *leuD* and *leuE* (A), and six candidate BCAA aminotransferase-encoding genes, *batA*, *batB*, *batC*, *batD*, *batE*, and *batF* (B), using cassettes that gene replaced the coding region with the A. fumigatus *pyrG* selectable marker (*AfpyrG^+^*) is shown. Download FIG S3, PDF file, 0.3 MB. Copyright © 2021 Steyer et al. 2021 Steyer et al. https://creativecommons.org/licenses/by/4.0/ This content is distributed under the terms of the Creative Commons Attribution 4.0 International license.

10.1128/mBio.00768-21.4 FIG S4 Complementation analysis of deleted genes. (A) Transformants of the *leuD*Δ mutant (RT411) with PCR-amplified wild-type *leuD* DNA were grown at 37°C for 2 days on solid supplemented ANM with (Leu) or without (–) 2 mM leucine and with 10 mM ammonium (NH_4_) or nitrate (NO_3_) as the nitrogen source. *leuD*^r^ indicates the reconstructed wild-type *leuD* by gene replacement of *leuDΔ::AfpyrG.* (B) Wild type (MH1), *leuD*Δ *leuE*Δ (RT444), and transformants of RT444 with a plasmid carrying wild-type *leuE* were grown at 37°C for 2 days on solid supplemented ANM with (Leu) or without (–) 2 mM leucine and with 10 mM ammonium (NH_4_) or nitrate (NO_3_) as the nitrogen source. Transformant #1 is a single-copy *leuE* integrant by homology via the *leuE*Δ flanking sequence, while transformants #2-#5 are multicopy transformants. (C) Southern analysis of NcoI-digested genomic DNA from *leuE* transformants, the *leuD*Δ *leuE*Δ recipient, and wild type, hybridized with the *leuE* plasmid insert as probe. (D) Single-copy homologous integration of the *leuE* plasmid into the *leuD*Δ *leuE*Δ recipient strain. Only integration via crossover at the left flank of *leuE* is shown. NcoI restriction sites and restriction fragment lengths are indicated. Sequences bound by the *leuE* probe are represented by the orange boxes. (E) Wild type (MH1), *batA*Δ *batB*Δ (RT457), and transformants of RT457 with PCR-amplified wild-type *batA* (*batA*^r^ *batB*Δ) or *batB* (*batA*Δ *batB*^r^) DNA, grown under ILV anabolic conditions at 37°C for 2 days on solid supplemented ANM with 10 mM nitrate (NO_3_) as the nitrogen source and combinations of 2 mM each isoleucine (I), leucine (L), and valine (V) to supplement potential auxotrophies. *batA*^r^ and *batB*^r^ indicate the reconstructed wild-type allele. (F) Strains from panel E grown under ILV catabolic conditions at 37°C for 2 days on solid supplemented ANM with 10 mM either isoleucine (Ile), leucine (Leu), or valine (Val) as the predominant nitrogen source and combinations of 2 mM each isoleucine (I), leucine (L), and valine (V) to supplement potential auxotrophies. In panels E and F, – represents an omitted amino acid. Download FIG S4, PDF file, 2.8 MB. Copyright © 2021 Steyer et al. 2021 Steyer et al. https://creativecommons.org/licenses/by/4.0/ This content is distributed under the terms of the Creative Commons Attribution 4.0 International license.

To complement the tight leucine auxotrophy of the *leuD*Δ *leuE*Δ double mutant, we introduced a plasmid carrying the wild-type *leuE* gene and directly selected transformants in the absence of leucine (Fig. S4B to D). Single-copy integration conferred partial leucine auxotrophy that resembled the *leuD*Δ single mutant, whereas multicopy transformants showed stronger growth, indicating that additional copies of the *leuE* gene partially suppress the *leuD*Δ phenotype. We next considered whether levels of expression were the source of the different degrees of effect of *leuD*Δ and *leuE*Δ. We found, using reverse transcription-quantitative PCR (RT-qPCR), that *leuD* had ∼64-fold higher expression than *leuE* after 16 h of growth in 10 mM ammonium-minimal medium. In transcriptome sequencing (RNA-seq) data from wild-type mycelia, *leuD* showed higher expression than *leuE* when grown on ammonium (35-fold), alanine (12-fold), and glutamine (13-fold) (Fig. 3B). As leucine production is regulated by feedback inhibition, we examined the effect of the *leuD*Δ mutation on expression of *leuE* and two other leucine biosynthesis genes, *luA* and *leuC*, by RT-qPCR, and *gdhA*, which is coregulated with leucine biosynthesis, using enzyme activity of LacZ expressed from the *gdhA-lacZ* translational fusion reporter gene (19, 27). For all three leucine biosynthesis genes, and for *gdhA-lacZ*, we found that *leuD*Δ resulted in increased expression over wild-type levels (Fig. 3C and D). Therefore, reduced leucine production as a result of *leuD*Δ results in compensation by upregulation of *leuE* and the other leucine biosynthesis genes as well as *gdhA*.

As *leuE*Δ had no effect on growth and *leuE* upregulation in the *leuD*Δ deletion mutant is expected to be LeuB dependent, we constructed a *leuB*Δ *leuD*Δ double mutant (Fig. 3A). In contrast to the *leuB*Δ and *leuD*Δ single mutants, which are leaky leucine auxotrophs, the *leuB*Δ *leuD*Δ double mutant is a strict leucine auxotroph, suggesting that LeuB regulation of *leuE* is required for leucine biosynthesis in the absence of *leuD.* We assayed *gdhA*-*lacZ* reporter gene expression in the double mutant (Fig. 3D). Unlike the single *leuD*Δ mutant, there was no increase in expression above *leuB*Δ levels in the double mutant, consistent with the *leuD*Δ-induced upregulation of leucine biosynthesis genes occurring through LeuB.

### Identification of six branched-chain amino acid aminotransferase genes.

The final step in leucine biosynthesis, catalyzed by the BCAA aminotransferase (BAT), is common to isoleucine and valine biosynthesis (Fig. 1). In S. cerevisiae, BAT enzymes are encoded by two genes, *BAT1* and *BAT2* (38, 39). Six BAT enzymes predicted to catalyze this step have been previously identified in A. nidulans (20). We confirmed the identity of these six BATs, and their coding genes, using BLASTP analysis and designated them BatA (AN4323), BatB (AN5957), BatC (AN7878), BatD (AN7876), BatE (AN0385), and BatF (AN8511). Pairwise protein sequence comparisons with Bat1p and Bat2p revealed >21% identity and >31% similarity to both proteins (Table 2). Alignment of these eight proteins showed strong conservation of NAD cofactor binding residues and absolute conservation of the catalytic lysine residue (Fig. S5). The two S. cerevisiae BATs function in different subcellular compartments. Bat1p is primarily targeted to mitochondria, whereas Bat2p is cytoplasmic (39). To predict the subcellular location of the six A. nidulans BAT enzymes, we used DeepLoc-1.0, TargetP v1.1, and Predotar targeting signal predictions (40–43). For all three algorithms, BatA and BatC, like Bat1p, were predicted to be predominantly mitochondrial, and the remaining BAT enzymes were predicted by DeepLoc-1.0 to localize in the cytoplasm (Data Set S1). The BAT protein alignment revealed that Bat1p, BatA, and BatC have extended N termini containing a predicted mitochondrial targeting signal (Fig. S5).

**TABLE 2:** Pairwise protein sequence comparisons of BATs

Protein^*a*^	Systematic name	Bat1p		Bat2p	
		% Identity	% Similarity	% Identity	% Similarity
Bat1p	YHR208W	100	100	73.5	81.2
Bat2p	YJR148W	73.5	81.2	100	100
BatA	AN4323	49.3	59.7	49.0	59.2
BatB	AN5957	40.5	54.4	43.4	58.6
BatC	AN7878	44.9	62.9	45.8	59.9
BatD	AN7876	24.3	41.3	23.8	41.4
BatE	AN0385	24.2	36.6	24.9	39.0
BatF	AN8511	21.7	31.8	25.1	35.8

a A. nidulans BatA, BatB, BatC, BatD, BatE, and BatF branched-chain amino acid aminotransferase full-length protein sequences were aligned pairwise and compared with S. cerevisiae Bat1p and Bat2p.

10.1128/mBio.00768-21.5 FIG S5 Clustal Omega alignment of putative BCAA aminotransferases. Clustal Omega alignment of Bat1p and Bat2p from S. cerevisiae with AN4323 (BatA), AN5957 (BatB), AN7878 (BatC), AN7876 (BatD), AN0385 (BatE), and AN8511 (BatF) from A. nidulans. The cofactor binding residues (green) and mitochondrial targeting signals (blue) are highlighted. The catalytic lysine is marked with a star above and below. Shading was performed with Boxshade with a minimum of 0.5 identity (black) or similarity (gray). Download FIG S5, PDF file, 0.9 MB. Copyright © 2021 Steyer et al. 2021 Steyer et al. https://creativecommons.org/licenses/by/4.0/ This content is distributed under the terms of the Creative Commons Attribution 4.0 International license.

10.1128/mBio.00768-21.9 DATA SET S1 Subcellular localization prediction for BATs using DeepLoc-1.0, TargetP v 1.1 and Predotar. Download Data Set S1, XLSX file, 0.04 MB. Copyright © 2021 Steyer et al. 2021 Steyer et al. https://creativecommons.org/licenses/by/4.0/ This content is distributed under the terms of the Creative Commons Attribution 4.0 International license.

We examined the colinearity of genes surrounding each of the six A. nidulans BAT-encoding genes to identify orthologous genes (Fig. S6). *batA* and *batB* orthologs are conserved in regions of high colinearity in all 27 species. *batE* orthologs are found in a region of moderate colinearity in 13 species. In contrast, *batC*, *batD*, and *batF* were located in regions lacking colinearity. *batD* only had orthologs in A. niger and A. oryzae, whereas *batC* and *batF* have no predicted ortholog. Interestingly, two of the BAT-encoding genes, *batC* and *batD*, are separated by just 2 kbp within the aspercryptins secondary metabolite gene cluster (44–47). The tight physical linkage of these two genes suggests that they arose from gene duplication by unequal crossover and, therefore, would show high sequence homology. However, the proteins encoded by these genes are highly diverged, showing only 28.9% protein sequence identity.

10.1128/mBio.00768-21.6 FIG S6 Colinearity of BCAA aminotransferase genes in aspergilli. The colinearity of syntenic regions for (A) AN4323 (*batA*), (B) AN5957 (*batB*), (C) AN7878 (*batC*), (D) AN7876 (*batD*), (E) AN0385 (*batE*), and (F) AN8511 (*batF*) was illustrated using the GBrowse genome browser of FungiDB with Aspergillus genomes displayed in the same order as in Fig. S2. Download FIG S6, PDF file, 0.8 MB. Copyright © 2021 Steyer et al. 2021 Steyer et al. https://creativecommons.org/licenses/by/4.0/ This content is distributed under the terms of the Creative Commons Attribution 4.0 International license.

**FIG 4:**
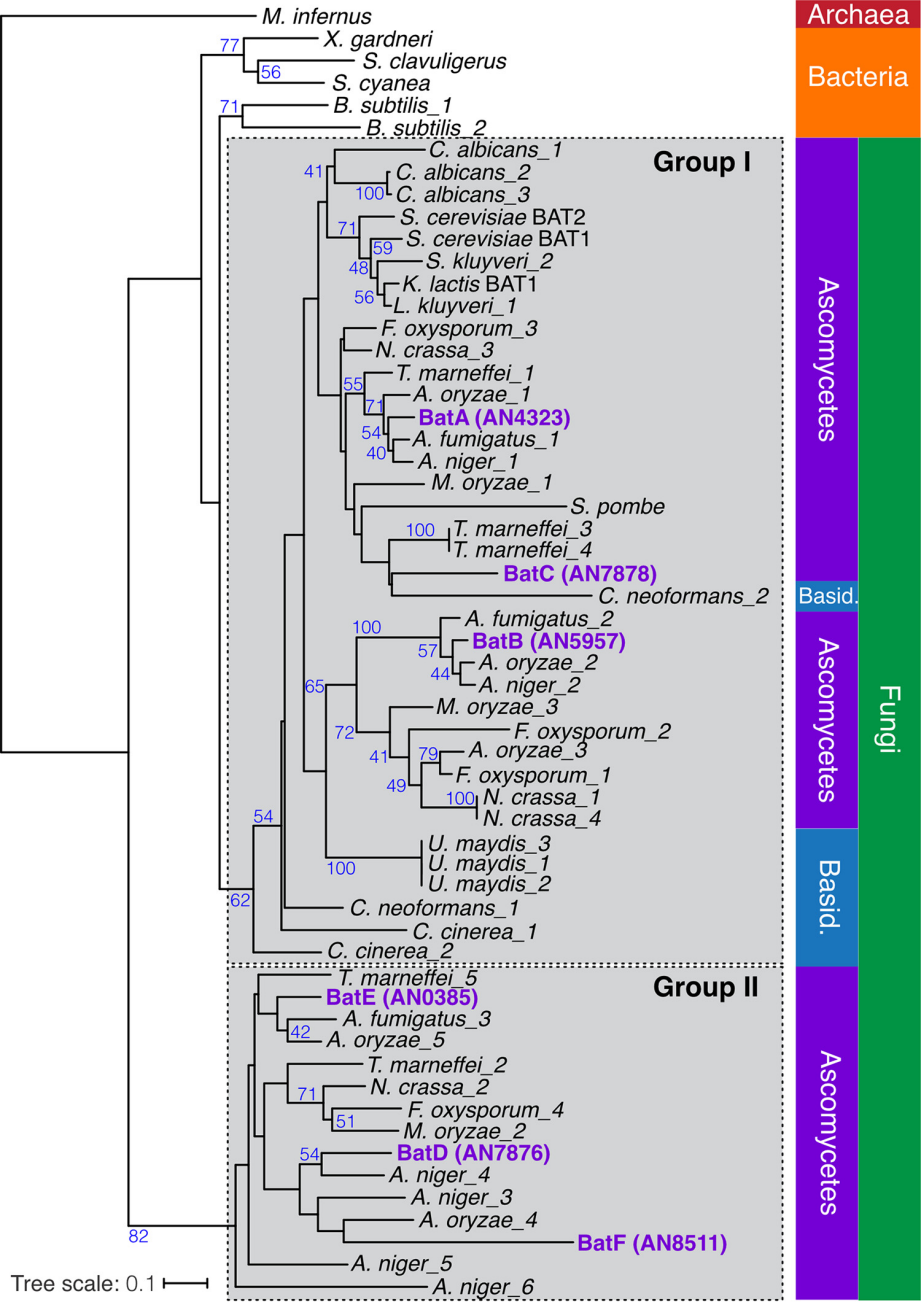
Phylogeny of BCAA aminotransferases. Unrooted phylogeny of BCAA aminotransferases. Bootstrap support (100 replicates) greater than 40% is shown. Protein sequences for aspergilli were downloaded from AspGD, sequences for S. cerevisiae were downloaded from SGD, and all other sequences came from Pfam or NCBI. *Archaea*, Methanocaldococcus infernus (D5VSZ6.1); *Bacteria*, Bacillus subtilis (1, O31461.1; 2, P39576.5), Streptomyces clavuligerus (B5H0M8.1), *S. cyanea* (H5XQS6.1), Xanthomonas gardneri (F0C966.1); Basidiomycota (Basid.), *C. cinerea* (1, A8N0B4.2; 2, A8N0V2.2), C. neoformans (1, Q5K761.1; 2, Q5KD20.1), *U. maydis* (1, XP_011386074.1; 2, NC_026478.1:289079-290305; 3, CM003140.1:289079-290305); Ascomycota, A. fumigatus (1, Afu4g06160; 2, Afu2g10420; 3, Afu1g01680), A. nidulans (BatA, AN4323; BatB, AN5957; BatC, AN7878; BatD, AN7876; BatE, AN0385; BatF, AN8511), A. niger (1, An04g00430; 2, An02g06150; 3, An09g01990; 4, An10g00620; 5, An01g06530; 6, An05g01100), A. oryzae (1, AO090023000977; 2, AO090011000598; 3, AO090023000123; 4, AO090011000044; 5, AO090005000936), C. albicans (1, Q59YS9.1; 2, Q5AHJ9.1; 3, Q5AHX4.1), Fusarium oxysporum (1, F9FH16.1; 2, F9FH71.1; 3, F9FL84.1; 4, F9FPH4.1), *M. oryzae* (1, G4MK83.1; 2, G4MNR9.1; 3, G4NDD5.1), N. crassa (1, Q9HEB7.2; 2, Q7SFT9.2; 3, Q7S699.1; 4, Q1K779.1), S. cerevisiae (Bat1p, YHR208W; Bat2p, YJR148), S. pombe (O14370.2), Kluyveromyces lactis (XP_451451.1), *Saccharomyces kluyveri* (1, CM000688.1:1070225-1071418; 2, CM000688.1:c1071418-1070225), and *Talaromyces marneffei* (1, XP_002144420.1; 2. XP_002147519.1; 3. XP_002148544.1; 4. XP_002148548.1; 5. XP_002152979.1). The scale bar corresponds to the branch length for an expected number of 0.1 substitutions per site. The two distinct fungal BAT clades are boxed.

To determine the relationship of the six A. nidulans BATs, we performed phylogenetic analysis (Fig. 4). The BATs formed two distinct groups within the fungi. Group I, the larger group containing 37 out of 52 of the fungal BATs, included BatA, BatB, BatC, and S. cerevisiae Bat1p and Bat2p, as well as at least one protein from every other fungus examined. Group II was a smaller group, with only 15 of the 52 proteins, and was almost entirely composed of BAT enzymes from Pezizomycotina genera (Aspergillus, *Penicillium*, Fusarium, *Neurospora*, *Magnaporthe*) and lacked any Saccharomycotina genera (*Saccharomyces*, *Candida*). Notably, BatC is in group I and BatD is in group II, consistent with separate recruitment to the aspercryptins cluster.

### Genetic analysis of six A. nidulans BATs.

**FIG 5:**
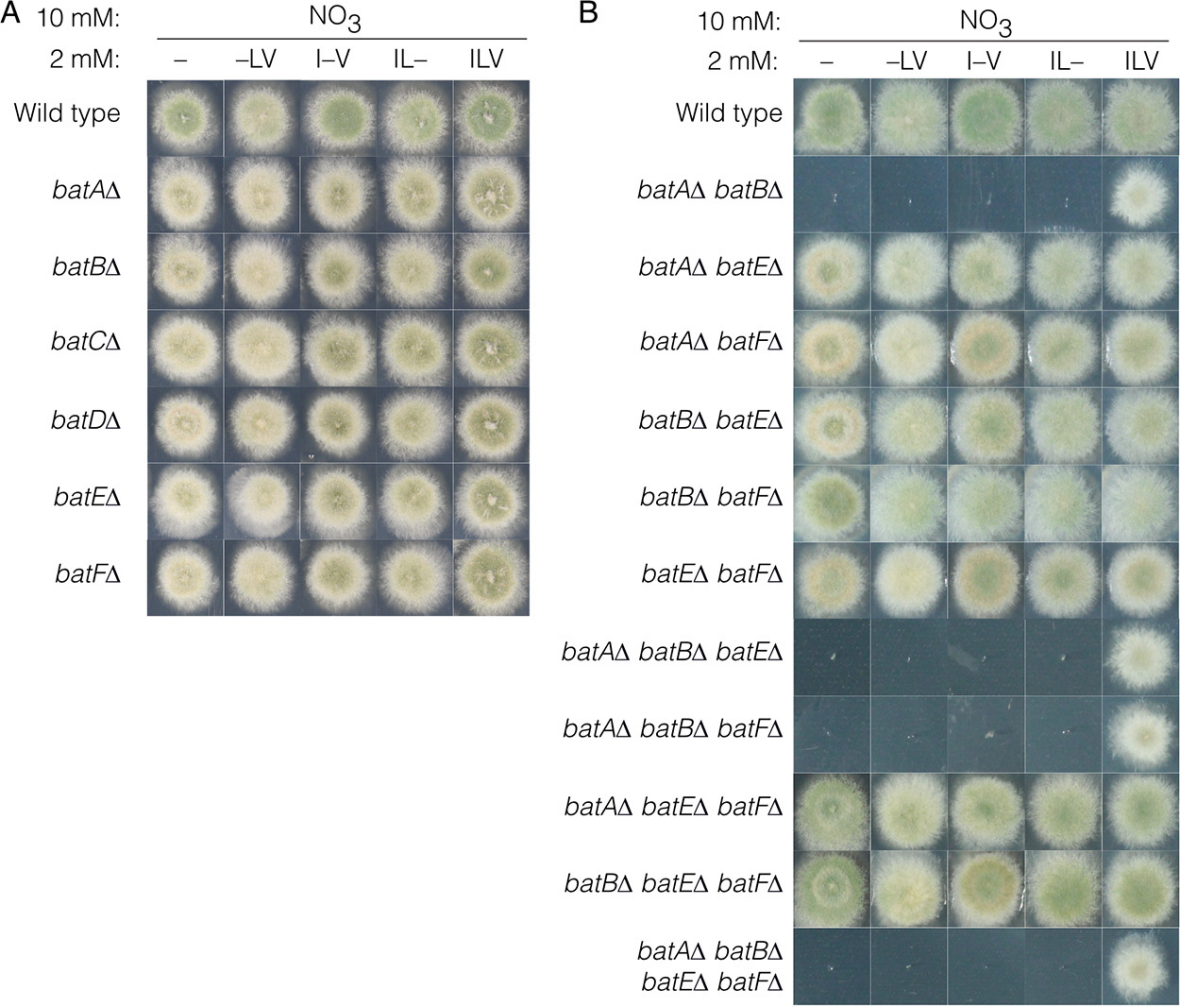
Single and combinatorial deletion analysis of BAT genes. (A) Wild-type (MH1), *batA*Δ (RT415), *batB*Δ (RT440), *batC*Δ (RT475), *batD*Δ (RT419), *batEΔ* (RT417), and *batF*Δ (RT441) strains were grown on supplemented ANM solid media for 2 days with 10 mM nitrate as the predominant nitrogen source and combinations of 2 mM (each) isoleucine (I), leucine (L), and valine (V) to supplement potential auxotrophies. –, an omitted amino acid. (B) Wild-type (MH1), *batA*Δ *batB*Δ (RT457), *batA*Δ *batE*Δ (RT648), *batA*Δ *batF*Δ (RT645), *batB*Δ *batE*Δ (RT636), *batB*Δ *batF*Δ (RT526), *batE*Δ *batF*Δ (RT466), *batA*Δ *batB*Δ *batE*Δ (RT520), *batA*Δ *batB*Δ *batF*Δ (RT523), *batA*Δ *batE*Δ *batF*Δ (RT647), *batB*Δ *batE*Δ *batF*Δ (RT531), and *batA*Δ *batB*Δ *batE*Δ *batF*Δ (RT642) strains grown on supplemented ANM solid media for 2 days with 10 mM nitrate (NO_3_) as the predominant nitrogen source and combinations of 2 mM each isoleucine (I), leucine (L), and valine (V) to supplement potential auxotrophies. –, an omitted amino acid.

The expansion of the number of BAT-encoding genes in A. nidulans indicates specialization for the production of isoleucine, leucine, or valine by specific BATs or the evolution of completely new roles. To determine which BAT-encoding genes were required for BCAA biosynthesis, we constructed individual knockout mutants of each of the six BATs (Fig. S3B; see Materials and Methods). Growth tests of the six individual *bat* knockout mutants showed none were BCAA auxotrophs (Fig. 5A). Therefore, each of the six BATs is dispensable for BCAA biosynthesis. During this study, the two BAT genes found in the aspercryptins gene cluster *batC* (AN7878) and *batD* (AN7876) were published by others as *atnH* and *atnJ*, respectively, and are thought to be involved in biosynthesis of 2-aminocaprylic acid, 2-aminododecanoic acid, and 2-aminodecanoic acid, three unusual BCAAs that are components of aspercryptins (46, 47).

**FIG 6:**
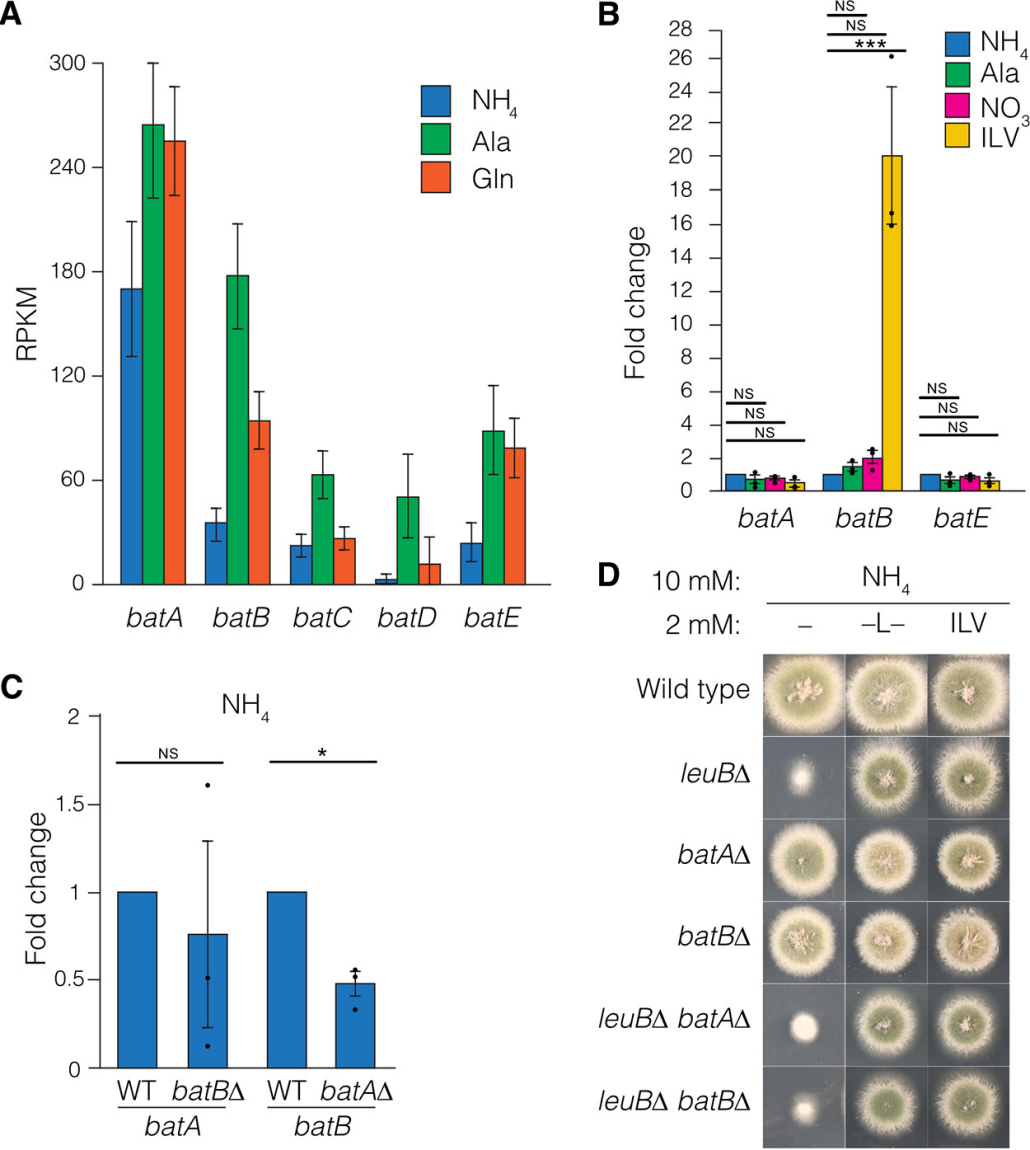
Expression analysis of BAT genes. (A) Mean reads per kilobase per million mapped reads (RPKM) from RNA-seq of MH1 grown at 37°C for 16 h in supplemented liquid ANM with 10 mM ammonium (NH_4_), glutamine (Gln), and alanine (Ala). Error bars depict SEM (*N* = 3). (B) RT-qPCR to measure expression levels of *batA*, *batB*, and *batE* under anabolic conditions compared with catabolic conditions. The wild type (MH1) was grown for 16 h in supplemented liquid ANM with 10 mM ammonium (NH_4_), nitrate (NO_3_), or alanine (Ala) (anabolic conditions) or 3.3 mM (each) ILV (catabolic conditions). Mean fold change (bars) in expression is shown relative to the wild type on 10 mM ammonium for three independent replicates (circles). ***, *P* ≤ 0.0001; NS, not significant, using a two-tailed Student's *t* test with equal variance. *batF* was not detected by either RNA-seq or RT-qPCR. (C) RT-qPCR of *batA* and *batB* in the wild-type (MH1), *batA*Δ (RT415), or *batB*Δ (RT440) strains grown for 16 h in supplemented liquid ANM with 10 mM ammonium. Mean fold change in expression (bars) relative to the wild type for three independent replicates (circles) is shown. *, *P* ≤ 0.05; NS, not significant, using a two-tailed Student's *t* test with equal variance. (D) Wild-type (MH1), *batA*Δ (RT415), *batB*Δ (RT440), *leuB*Δ (RT453), *leuB*Δ *batA*Δ (RT793), and *leuBΔ batBΔ* (RT794) strains were grown on supplemented ANM solid media for 2 days with 10 mM ammonium as the predominant nitrogen source with (ILV) or without (–) 2 mM (each) isoleucine, leucine, and valine or with 2 mM leucine (L).

Analysis of RNA-seq expression data from wild-type mycelia grown on ammonium, alanine, or glutamine (Fig. 6A) showed that *batA* has the highest expression under all three conditions. *batB* was the next most highly expressed and showed increased expression on alanine and glutamine compared to ammonium. *batC*, *batD*, and *batE* all showed intermediate expression levels, whereas *batF* was not expressed under these conditions. As *batC* and *batD* are involved in biosynthesis of unusual BCAAs (46, 47), we focused on the other four BAT genes. We measured expression of *batA*, *batB*, *batE*, and *batF* using RT-qPCR of RNA prepared from samples grown on ammonium, alanine, or nitrate. *batA*, *batB*, and *batE* expression did not substantially change under these conditions (Fig. 6B). *batF* was not expressed under these conditions, consistent with it being undetectable by RNA-seq. We constructed double, triple, and quadruple mutants combining *batA*Δ, *batB*Δ, *batE*Δ, and *batFΔ* by meiotic crossing. The *batA*Δ *batB*Δ double mutant, which combined deletions of the two most related and highly expressed genes, was a strict BCAA auxotroph and could only grow if supplemented with all three BCAAs (Fig. 5B). Therefore, BatA and BatB are the major BAT enzymes for isoleucine, leucine, and valine (ILV) biosynthesis. The *batA*Δ *batB*Δ *batE*Δ and *batA*Δ *batB*Δ *batF*Δ triple mutants and the *batA*Δ *batB*Δ *batE*Δ *batFΔ* quadruple mutant showed BCAA auxotrophy identical to that of the *batA*Δ *batB*Δ double mutant. In contrast, all of the other double and triple mutants constructed, which contained a wild-type copy of either *batA* or *batB*, were BCAA prototrophs. We confirmed that introduction of either the *batA* or *batB* gene into the *batA*Δ *batB*Δ mutant restored BCAA prototrophy (Fig. S4E). We investigated whether loss of either *batA* or *batB* would cause a compensatory increase in expression of *batB* or *batA*, respectively. However, on ammonium, *batA* expression was not upregulated in the *batB*Δ mutant and *batB* expression was not upregulated in the *batA*Δ mutant (Fig. 6C). This indicates that the expression levels of either one of the major *bat* genes for BCAA biosynthesis is sufficient for prototrophy. We constructed *leuB*Δ *batA*Δ and *leuB*Δ *batB*Δ double mutants. These two double mutants showed leaky leucine auxotrophy similar to that of the *leuB*Δ single mutant, indicating that *leuB*Δ is epistatic to *batA*Δ and *batB*Δ (Fig. 6D).

**FIG 7:**
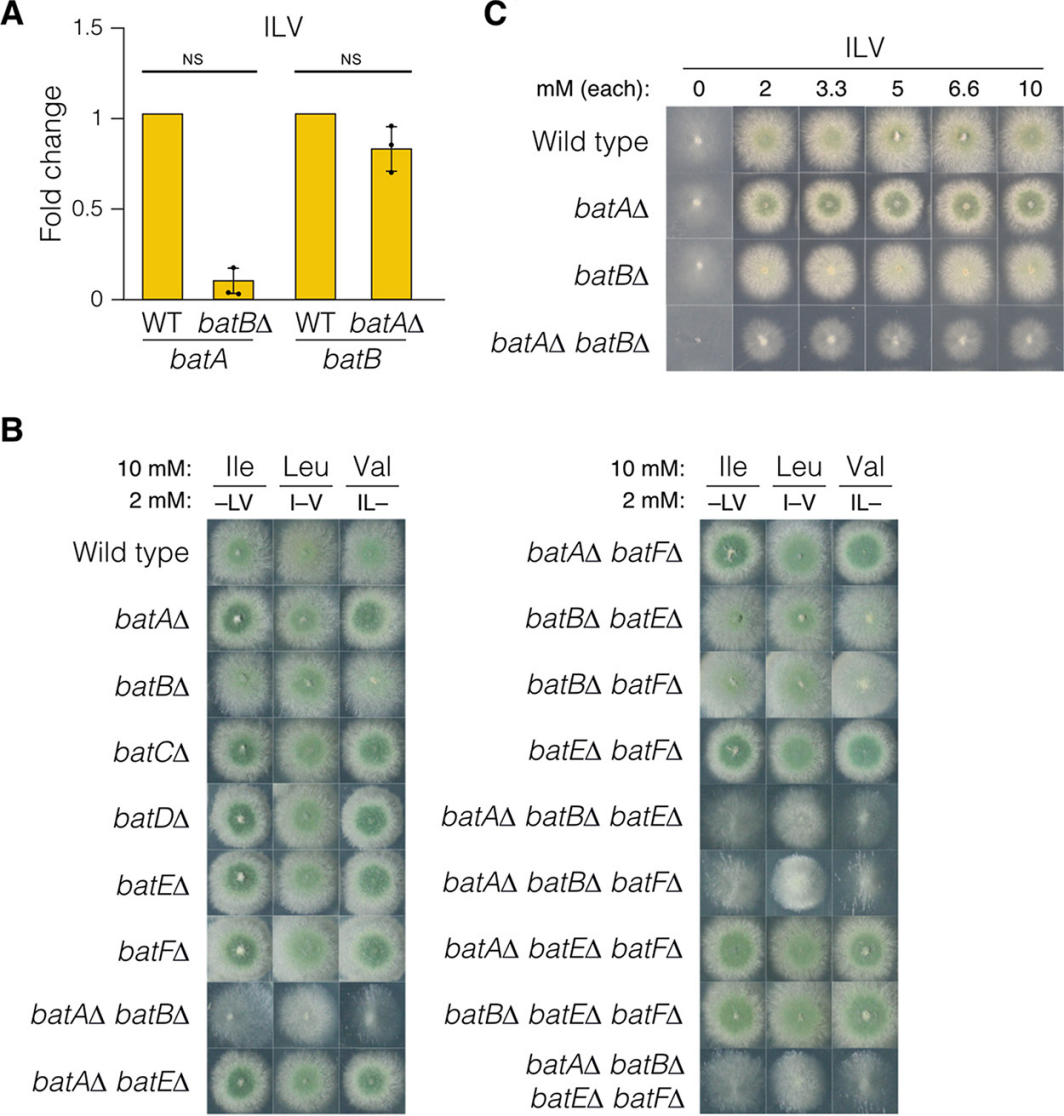
Combinatorial analysis of BAT genes during catabolic growth. (A) RT-qPCR of *batA* and *batB* in the wild-type (MH1), *batA*Δ (RT415), or *batB*Δ (RT440) strains grown for 16 h in supplemented liquid ANM with 3.3 mM (each) isoleucine (I), leucine (L), and valine (V), i.e., catabolic conditions. Mean fold change in expression (bars) relative to the wild type for three independent replicates (circles) is shown. NS, not significant using two-tailed Student's *t* test with equal variance. (B) Wild-type (MH1), *batA*Δ (RT415), *batB*Δ (RT440), *batC*Δ (RT475), *batD*Δ (RT419), *batEΔ* (RT417), *batF*Δ (RT441), *batA*Δ *batB*Δ (RT457), *batA*Δ *batE*Δ (RT648), *batA*Δ *batF*Δ (RT645), *batB*Δ *batE*Δ (RT636), *batB*Δ *batF*Δ (RT526), *batE*Δ *batF*Δ (RT466), *batA*Δ *batB*Δ *batE*Δ (RT520), *batA*Δ *batB*Δ *batF*Δ (RT523), *batA*Δ *batE*Δ *batF*Δ (RT647), *batB*Δ *batE*Δ *batF*Δ (RT531), and *batA*Δ *batB*Δ *batE*Δ *batF*Δ (RT642) strains were grown on supplemented ANM solid media for 2 days with 10 mM isoleucine (Ile), leucine (Leu), or valine (Val) as the predominant nitrogen source and combinations of 2 mM each isoleucine (I), leucine (L), and valine (V) to supplement auxotrophies. –, an omitted amino acid. (C) Wild-type (MH1), *batA*Δ (RT415), *batB*Δ (RT440), and *batA*Δ *batB*Δ (RT457) strains were grown on supplemented ANM solid media for 2 days at 37°C with increasing equimolar concentrations of isoleucine (I), leucine (L), and valine (V).

In addition to their role in BCAA biosynthesis, BATs also form the first step in ILV catabolism (28). We examined expression of *batA*, *batB*, *batE*, and *batF* with ILV as the sole nitrogen source to determine their expression pattern during catabolic conditions (Fig. 6B). For both *batA* and *batE*, expression levels were similar under anabolic and catabolic conditions. However, *batB* levels were elevated substantially during ILV catabolism compared with biosynthetic growth conditions, suggesting that BatB is the predominant catabolic enzyme. *batF* expression was undetectable. During BCAA catabolic growth, neither *batA* nor *batB* expression showed compensatory upregulation in the *batB*Δ or *batA*Δ strain, respectively (Fig. 7A). We assessed whether mutants carrying single or multiple BAT gene deletions could utilize each BCAA as the predominant nitrogen source in the presence of lower levels of the other two BCAAs to supplement the auxotrophy (Fig. 7B). All six single BAT mutants could utilize the three BCAAs. Mutants lacking *batB* but not *batA* showed slightly reduced colony morphology compared with *batB*^+^ strains. Notably, mutants lacking both *batA* and *batB* showed severely reduced growth on each of the BCAAs as a predominant nitrogen source, and the reduction in growth was greater on isoleucine and valine than on leucine. We also examined growth of the *batA*Δ and *batB*Δ single and double mutants on increasing concentrations of equimolar ILV and found that *batB*Δ shows reduced colony morphology compared with both wild-type and *batA*Δ strains but stronger growth than the *batA*Δ *batB*Δ double mutant (Fig. 7C). Therefore, BatA and BatB are the major BAT enzymes in A. nidulans for both BCAA biosynthesis and utilization. We did not observe a phenotype for *batE*Δ or *batF*Δ mutant in BCAA catabolism. Transformation analysis of the *batA* or *batB* gene into the *batA*Δ *batB*Δ recipient repaired BCAA utilization to the wild-type phenotype (Fig. S4F).

### Regulation of leucine biosynthesis pathway gene expression by LeuB.

**FIG 8:**
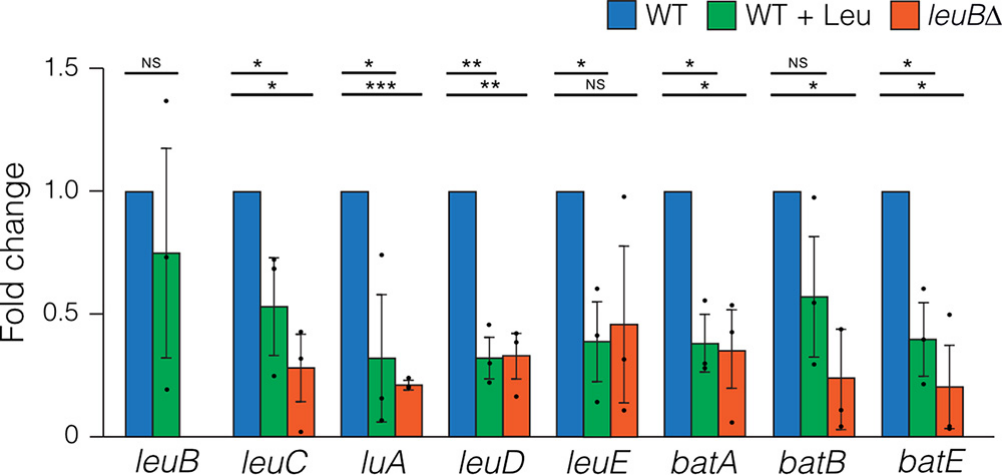
LeuB regulation of the leucine biosynthesis genes. RT-qPCR of BCAA biosynthesis genes from wild-type (MH1) and *leuBΔ* (MH12609) strains grown for 16 h in supplemented liquid ANM-10 mM ammonium with or without 2 mM leucine (Leu). Expression is relative to the wild type. The means (bars) and individual results from three independent replicates (circles) are shown. *, *P* ≤ 0.05; **, *P* ≤ 0.001; ***, *P* ≤ 0.0001; NS, not significant, using two-tailed Student's *t* test with equal variance.

The transcription factor LeuB is thought to regulate leucine biosynthesis genes because the *leuB*Δ mutant is a leaky leucine auxotroph (19). To determine whether LeuB regulates these genes in response to leucine levels, we performed RT-qPCR on RNA isolated from mycelia grown with exogenous leucine, which represses LeuB activation (27), and in a *leuB*Δ strain (Fig. 8). *leuB* expression was not altered in response to leucine. The six genes we demonstrated to function in leucine biosynthesis, *leuC*, *luA*, *leuD*, *leuE*, *batA*, and *batB*, as well as *batE*, showed decreased expression in response to exogenous leucine and/or in the *leuB*Δ mutant compared to the wild type.

## DISCUSSION

We have completed annotation of the A. nidulans leucine biosynthesis pathway and characterized the genes encoding enzymes for the final two steps. Our analysis has revealed divergence between aspergilli and yeast in the number of genes encoding the enzymes for each step. In S. cerevisiae, ketoisovalerate is converted to α-IPM by two α-IPM synthetases, Leu4p and Leu9p, which form homodimers and heterodimers that show differential sensitivity to leucine feedback inhibition (23–26, 31). In contrast, a single α-IPM synthetase gene exists in A. nidulans (27). α-IPM is converted to β-IPM by the isopropylmalate isomerase, which is encoded by a single gene in both S. cerevisiae (*LEU1*) and A. nidulans (*luA*) (19, 21). β-IPM is then converted to ketoisocaproate by a single β-IPM dehydrogenase in S. cerevisiae, Leu2p (35, 36), but two enzymes, LeuD and LeuE, in A. nidulans. The final step in BCAA biosynthesis is catalyzed by BCAA aminotransferase (BAT). S. cerevisiae has two BAT genes (38, 39). A. nidulans carries six BAT genes; however, primarily two, BatA and BatB, play major roles in ILV biosynthesis. Interestingly, the genes encoding the dimeric enzymes in the pathway, α-IPM synthetase (31), β-IPM dehydrogenase (48, 49), and BAT (50), differ in copy number, whereas the nonduplicated gene for α-IPM isomerase is monomeric (51). The *LEU4/LEU9* and *BAT1/BAT2* gene duplications resulted from the ancestral whole-genome duplication (WGD) and exhibit functional diversification associated with the acquisition of fermentative metabolism (52).

The Aspergillus lineage did not experience an ancestral WGD, but alternative mechanisms have mediated gene duplication within the leucine biosynthesis pathway. The acquisition of additional copies of genes often leads to robustness via the evolution of new functions but in some cases confers fragility (52–54). We found that both *leuD* and *leuE* function in leucine biosynthesis, although *leuE* plays a lesser role based upon its low expression, the prototrophy of the *leuE*Δ mutant, and the leaky leucine auxotrophy conferred by deletion of *leuD*. This gene duplication provides robustness in the form of redundancy, as perturbation of leucine biosynthesis by deletion of *leuD* resulted in LeuB-dependent upregulation of *leuE* and partial compensation of the leucine auxotrophy. Our functional analysis showed that each of the six A. nidulans BATs are dispensable. Combining BAT gene deletions, however, revealed that BatA and BatB are the major enzymes in both BCAA biosynthesis and utilization. BatA contains a mitochondrial targeting signal and shows higher biosynthetic expression, while the likely cytoplasmic BatB shows higher catabolic expression. Therefore, BatA and BatB are equivalent to mitochondrial and predominantly biosynthetic Bat1p and cytoplasmic and predominantly catabolic Bat2p in S. cerevisiae (50, 55, 56). BatA and BatB show redundancy in both biosynthesis and catabolism. BAT function is also distributed between two paralogs in *Lachancea kluyveri*, with one major biosynthetic BAT and both involved in aerobic metabolism (57). In contrast, Kluyveromyces lactis has just one BAT gene, which encodes a bifunctional enzyme for BCAA biosynthesis and degradation, and this is thought to be the ancestral type prior to the WGD and subfunctionalization of Bat1p and Bat2p in S. cerevisiae (50).

The dispensability of *batC*, *batD*, *batE*, and *batF* for BCAA biosynthesis and catabolism suggests evolution of novel roles. We showed that *batE* is regulated by leucine and LeuB, similar to other leucine biosynthesis genes, but expression levels are low and we did not observe a phenotype for the *batE*Δ mutant. However, *batE* expression is induced during hypoxia in the absence of glucose-to-ethanol fermentation, in association with elevated BCAA biosynthesis that occurs as a mechanism to generate NAD^+^ and survive anaerobic stress (20, 58). BatE does not appear to contribute to BCAA metabolism under our normoxic growth conditions but may play a role during anaerobic stress. *batC* (*atnH*) and *batD* (*atnJ*) are members of the aspercryptin biosynthetic gene cluster, with presumed roles in transamination of the unusual BCAAs 2-aminocaprylic acid, 2-aminodecanoic acid, and 2-aminododecanoic acid (46, 47). Aspercryptins contain three BCAAs (isoleucine or valine, 2-aminocaprylic acid, and 2-aminododecanoic or 2-aminodecanoic acid). Expression of *batF* was undetectable under our growth conditions or growth conditions used for RNA-seq by others (59). *batF* is adjacent to the terriquinone A (*tdi*) biosynthetic gene cluster and may also be associated with secondary metabolism (45, 60–62).

Regulation of leucine biosynthesis is best understood in S. cerevisiae where both activation and repression are mediated by the Zn(II)_2_Cys_6_ transcription factor Leu3p (16). When leucine is abundant, it interacts with α-IPM synthetase, inhibiting its function, which decreases cellular α-IPM levels and leads to Leu3p acting as a repressor (16, 24). When leucine levels decrease, α-IPM synthetase is not inhibited, and α-IPM interacts with Leu3p, causing a conformational change and resulting in Leu3p switching to an activator (16, 30). We observed repression by exogenous leucine in wild-type cells of all six genes that function in leucine biosynthesis, *luA*, *leuC*, *leuD*, *leuE*, *batA*, and *batB*, as well as *batE*, indicating this feedback mechanism operates in A. nidulans. Deletion of the A. nidulans *LEU3* ortholog *leuB* confers leaky leucine auxotrophy (19), which we have now shown is due to decreased expression of the leucine biosynthesis genes. The *leuD*Δ mutant shows leaky leucine auxotrophy and increased expression of other leucine biosynthesis genes, which likely results from reduced cellular levels of the negative feedback mediator leucine and increased α-IPM inducer levels due to increased β-IPM levels increasing the reverse reaction rate carried out by the bidirectional α-IPM isomerase encoded by *luA*.

The absence of the leucine biosynthesis pathway in animals and the reduced virulence of leucine auxotrophs (4–6, 9, 33, 97) render leucine biosynthesis enzymes strong candidate targets for antifungals. Our studies of the genes in this pathway indicate that the feedback mechanisms and gene duplications present in the aspergilli must be considered in target selection to avoid increased LeuB-dependent expression of other leucine biosynthesis genes in response to an antifungal agent targeting this pathway. The strongest target would be α-IPM synthetase (LeuC), as reduced activity of this enzyme leads to decreased α-IPM and repression of leucine biosynthesis genes by LeuB (27). The benefit of targeting this step would be in cross regulation of nitrogen assimilation by reduced expression of *gdhA* and potentially reduced cellular glutamate and glutamine levels.

Overall, this study has completed the annotation of the genes required for leucine biosynthesis in A. nidulans and demonstrated regulation of the pathway genes by LeuB. We have found roles for *leuD* and *leuE* in leucine biosynthesis and for *batA* and *batB* in BCAA biosynthesis and catabolism. Roles for *batC* (*atnH*) and *batD* (*atnJ*) in aspercryptins production have now been reported (46, 47), but the roles of *batE* and *batF* remain to be determined.

## MATERIALS AND METHODS

### A. nidulans strains, media, and genetic analysis.

A. nidulans strains and genotypes are listed in Table 3 using conventional nomenclature (98). A. nidulans growth conditions and media were as described previously (63, 64). Aspergillus nitrogen-free minimal medium (ANM), pH 6.5, containing 1% (wt/vol) glucose as the sole carbon source, was supplemented for auxotrophies and nitrogen sources (10 mM final concentration), unless otherwise stated. A. nidulans growth testing and genetic analysis were as described previously (64).

**TABLE 3:** Strains used in this study

Strain	Origin	Genotype^*a*^
MH1	M. J. Hynes	*biA1*
MH10865	R. B. Todd	*yA1 pabaA1 pyrG89 argB::fmdS-lacZ areAΔ(5′)*::*riboB*
MH11068	M. J. Hynes	*pyrG89 pyroA4 nkuAΔ*::*Bar*
MH12609	M. A. Davis	*yA1 pabaA1 leuBΔ*::*riboB pyroA4 nkuAΔ*::*Bar niiA4*
MH12181	Downes et al. (27)	*leuBΔ*::*riboB amdS*::*AfpyroA-gdhA*(−753 bp)*-lacZ pyroA4 niiA4*
RT411	Transformant of MH11068	*pyrG89 pyroA4 nkuAΔ*::*Bar leuDΔ*::*AfpyrG*
RT412	RT411 × MH10865	*yA1 pabaA1 pyrG89 leuDΔ*::*AfpyrG*
RT413	Transformant of MH11068	*pyrG89 pyroA4 nkuA*Δ::*Bar leuEΔ*::*AfpyrG*
RT414	RT413 × MH10865	*yA1 pabaA1 pyrG89 leuEΔ*::*AfpyrG*
RT415	Transformant of MH11068	*pyrG89 batAΔ*::*AfpyrG pyroA4 nkuA*Δ::*Bar*
RT416	RT415 × MH10865	*yA1 pyrG89 pabaA1 batAΔ*::*AfpyrG*
RT417	Transformant of MH11068	*pyrG89 pyroA4 nkuAΔ*::*Bar batEΔ*::*AfpyrG*
RT418	RT417 × MH10865	*yA1 pabaA1 pyrG89 batEΔ*::*AfpyrG*
RT419	Transformant of MH11068	*pyrG89 batDΔ*::*AfpyrG pyroA4 nkuAΔ*::*Bar*
RT440	Transformant of MH11068	*pyrG89 batBΔ*::*AfpyrG pyroA4 nkuAΔ*::*Bar*
RT441	Transformant of MH11068	*pyrG89 pyroA4 nkuAΔ*::*Bar batFΔ*::*AfpyrG*
RT444	RT411 × RT414	*pyrG89 pyroA4 leuEΔ*::*AfpyrG leuDΔ*::*AfpyrG*
RT452	MH12181 × MH11068	*pyrG89 leuBΔ*::*riboB amdS*::*AfpyroA-gdhA*(−753 bp)*-lacZ pyroA4*
RT453	MH12181 × MH11068	*pyrG89 leuBΔ*::*riboB amdS*::*AfpyroA-gdhA*(−753 bp)*-lacZ pyroA4 niiA4*
RT454	RT418 × RT419	*yA1 pabaA1 pyrG89 batDΔ*::*AfpyrG batEΔ*::*AfpyrG*
RT457	RT416 × RT440	*pyrG89 batBΔ*::*AfpyrG batAΔ*::*AfpyrG pyroA4*
RT458	RT412 × RT453	*yA1 pabaA1 pyrG89 amdS*::*AfpyroA-gdhA*(−753 bp)*-lacZ leuDΔ*::*AfpyrG*
RT460	RT412 × RT453	*yA1 pabaA1 pyrG89 leuBΔ*::*riboB amdS*::*AfpyroA-gdhA*(−753 bp)*-lacZ leuDΔ*::*AfpyrG*
RT462	RT412 × RT453	*leuBΔ*::*riboB amdS*::*AfpyroA-gdhA*(−753 bp)*-lacZ leuDΔ*::*AfpyrG niiA4*
RT466	RT441 × RT454	*pabaA1 pyrG89 batFΔ*::*AfpyrG batEΔ*::*AfpyrG*
RT475	Transformant of MH11068	*pyrG89 batCΔ*::*AfpyrG pyroA4 nkuAΔ*::*Bar*
RT520	RT457 × RT466	*batB*Δ::*AfpyrG batA*Δ::*AfpyrG batE*Δ::*AfpyrG*
RT523	RT457 × RT466	*pabaA1 batB*Δ::*AfpyrG batA*Δ::*AfpyrG batF*Δ::*AfpyrG*
RT524	RT457 × RT466	*pabaA1 batB*Δ::*AfpyrG batA*Δ::*AfpyrG batE*Δ::*AfpyrG*
RT525	RT457 × RT466	*batB*Δ::*AfpyrG pyroA4 batF*Δ::*AfpyrG batE*Δ::*AfpyrG*
RT526	RT457 × RT466	*batB*Δ::*AfpyrG batF*Δ::*AfpyrG*
RT531	RT457 × RT466	*batB*Δ::*AfpyrG batF*Δ::*AfpyrG batE*Δ::*AfpyrG*
RT636	RT525 × RT524	*batB*Δ::*AfpyrG batE*Δ::*AfpyrG*
RT642	RT525 × RT523	*batB*Δ::*AfpyrG batA*Δ::*AfpyrG pyroA4 batF*Δ::*AfpyrG batE*Δ::*AfpyrG*
RT645	RT415 × RT466	*pabaA1 batA*Δ::*AfpyrG pyroA4 batF*Δ::*AfpyrG*
RT647	RT415 × RT466	*pabaA1 batA*Δ::*AfpyrG batF*Δ::*AfpyrG batE*Δ::*AfpyrG*
RT648	RT415 × RT466	*batA*Δ::*AfpyrG pyroA4 batE*Δ::*AfpyrG*
RT793	RT453 × RT415	*pyrG89 batA*Δ::*AfpyrG leuBΔ*::*riboB pyroA4 nkuAΔ*::*Bar niiA4*
RT794	RT453 × RT440	*pyrG89 batB*Δ::*AfpyrG leuBΔ*::*riboB pyroA4 nkuAΔ*::*Bar niiA4*

a All strains carry *veA1*.

### Standard molecular techniques.

Escherichia coli NM522 cells [F′ *proA*^+^*B*^+^ *lacI*^q^ Δ(*lacZ*)M15/Δ(*lac-proAB*) *glnV thi-1* Δ(*hsdS-mcrB*)*5*] (65) were employed for molecular cloning (66). Plasmid DNA was isolated using the Wizard Plus SV miniprep DNA purification kit (Promega). A. nidulans genomic DNA was isolated according to reference 67. PCR products and DNA fragments isolated from agarose gels were cleaned with the Wizard SV gel and PCR clean-up system (Promega). Restriction enzyme digestions (Promega, New England Biolabs), dephosphorylation with Arctic shrimp alkaline phosphatase (Promega), and ligations using T4 DNA ligase (Promega) followed the manufacturers’ instructions. DNA was separated on 1 to 2% agarose gels by electrophoresis in 1× Tris-acetate-EDTA (TAE) buffer. PCRs used *Ex Taq* (TaKaRa), Phusion (Finnzymes), or AccuStart II Geltrack PCR supermix (Quanta Biosciences) DNA polymerases according to the manufacturers’ instructions, with 1 ng plasmid or 100 ng A. nidulans genomic DNA templates. All reactions followed recommended denaturing and annealing conditions with 33 to 36 amplification cycles. Oligonucleotide PCR primers (Integrated DNA Technologies) are described in Table S1 in the supplemental material. DNA sequencing to confirm correct amplifications and cloning was performed at the Kansas State University DNA Sequencing and Genotyping Facility. Southern hybridizations used either Hybond N+ or Hybond XL membranes (GE Healthcare) and the DIG (digoxigenin) high prime DNA labeling and detection starter kit II (Roche) by following the manufacturer’s instructions.

10.1128/mBio.00768-21.7 TABLE S1 Leucine biosynthesis genes genomic PCR primer sets. Download Table S1, PDF file, 0.02 MB. Copyright © 2021 Steyer et al. 2021 Steyer et al. https://creativecommons.org/licenses/by/4.0/ This content is distributed under the terms of the Creative Commons Attribution 4.0 International license.

### Strain construction.

A. nidulans transformation was performed as described previously (27) using the *nkuA*Δ nonhomologous integration-defective mutant for targeted integration (68). Knockout constructs, generated by the A. nidulans whole-genome gene deletion constructs program (69), were sourced from the Fungal Genetics Stock Center, Manhattan, KS (70), and were transformed into MH11068 (*pyrG89 nkuA*Δ::*Bar*) and selected for uracil and uridine prototrophy to generate *leuDΔ* (AN0912Δ; RT411, Δ−7 to +1,431 bp), *leuE*Δ (AN2793Δ; RT413, Δ−6 to +1,233 bp), *batA*Δ (AN4323; RT415, Δ+65 to +1,722 bp), *batB*Δ (AN5957Δ; RT440, Δ+25 to +1,395 bp), *batC*Δ (AN7878Δ; RT475, Δ−10 to +1,222 bp), *batD*Δ (AN7876Δ; RT419, Δ−7 to +1,297 bp), *batE*Δ (AN0385Δ; RT417, Δ+27 bp to 1,302 bp), and *batF*Δ (AN8511; RT441, Δ−9 to +1,230 bp) strains. Selection media for *leuD*Δ and *leuE*Δ transformants were supplemented with 2 mM leucine, and BAT gene deletion transformants were supplemented with ILV (2 mM each). The Aspergillus fumigatus *pyrG* (*AfpyrG*) marker showed position effect in the *batC*Δ mutant, incompletely complementing the pyrimidine auxotrophy of the *pyrG89* mutation. Full complementation of *pyrG89* by *AfpyrG* was observed in the other deletion mutants generated in this study. Pyrimidine supplementation was used in all growth tests. All deletion mutants were confirmed by Southern blotting as a single homologous double-crossover integration at the correct locus by probing with the 982-bp KpnI-SspI fragment of *AfpyrG^+^* (data not shown). Meiotic crossing was used to generate double, triple, and quadruple mutants. The presence of each deletion in the progeny of crosses was confirmed by diagnostic Southern blotting or diagnostic PCR. The *leuD*Δ mutant was repaired by introduction of a wild-type *leuD* PCR product (−960 to +3216) amplified from MH1, with direct selection for simultaneous resistance to 1 mg ml^−1^ 5-fluoroorotic acid (5-FOA) in the absence of exogenous leucine. The *leuD*Δ *leuE*Δ mutant was complemented with the plasmid pJS249, which carries *leuE* (−913 to +2877) PCR amplified from MH1 and cloned into pGEMTeasy by transformation with direct selection for leucine prototrophy. The *batA*Δ *batBΔ* double mutant was complemented with the wild-type *batA* (−717 to +2558) or *batB* (−725 to +2187) gene using plasmids (pJS244 and pJS255, respectively) containing PCR-amplified DNA from MH1 cloned into pGEMTeasy. Transformants were directly selected for growth in the absence of exogenous ILV.

### β-Galactosidase assays.

β-Galactosidase assays were performed as described previously (71) using soluble protein extracts. β-Galactosidase specific activity is defined as *A*_420_ × 10^3^ min^−1 ^mg^−1^ of soluble protein. Protein concentrations were determined using Bio-Rad assay reagent (Bio-Rad).

### RNA preparation.

Total RNA was isolated by grinding mycelia under liquid nitrogen and subsequent addition to RNA extraction buffer (7.0 M urea, 100 mM Tris-HCl, pH 8.0, 10 mM EDTA, 1.0% sodium dodecyl sulfate) followed by two phenol-chloroform-isoamyl alcohol extractions and one chloroform extraction (66). RNA was precipitated in 3 M ammonium acetate and 50% isopropanol, resuspended in diethyl pyrocarbonate-H_2_O, and reprecipitated overnight in 4 M lithium chloride at −20°C. RNA quality was determined by visualization after electrophoretic separation in a 1.2% agarose gel containing 1.1% formaldehyde run in 1× morpholinepropanesulfonic acid (MOPS) buffer (20 mM MOPS, pH 7.0, 5 mM sodium acetate, 1 mM EDTA). RQ1 DNase (Promega) treatment of RNA followed the manufacturer’s instructions.

### RT-qPCR.

For reverse transcriptase-quantitative PCR (RT-qPCR), cDNA was produced using the reverse transcriptase system (Promega) or qScript cDNA supermix (Quanta Biosciences). RT-qPCR used a MyiQ thermocycler (Bio-Rad) with iTAQ universal SYBR green supermix (Bio-Rad), and results were analyzed with iQ5 v2.1 (Bio-Rad). Fold change was calculated using the ΔΔ*C_T_* method with β-tubulin-encoding *benA* as the reference gene (72–74). Primers (IDT) were designed to specifically amplify cDNA by overlapping a splice junction. Primer sequences used for RT-qPCR, target regions, and efficiencies are listed in Table S2.

10.1128/mBio.00768-21.8 TABLE S2 Leucine biosynthesis gene RT-qPCR primers. Download Table S2, PDF file, 0.03 MB. Copyright © 2021 Steyer et al. 2021 Steyer et al. https://creativecommons.org/licenses/by/4.0/ This content is distributed under the terms of the Creative Commons Attribution 4.0 International license.

### RNA-seq.

PolyA^+^ RNA, isolated from three independent biological replicates of wild-type (MH1) mycelia grown for 16 h in supplemented liquid ANM with 10 mM ammonium, glutamine, or alanine, was fragmented to 180 bp and indexed using the TruSeq stranded total RNA sample preparation kit (Illumina). Multiplexed libraries were sequenced using 50-bp single-end reads on the Illumina Hi-Seq 2500 system (Kansas University Medical Center Genome Sequencing Facility, Kansas City, KS). RNA-seq analysis was conducted using Galaxy (www.galaxyproject.org) (75–77). Reads were processed with FASTQ Groomer (78) and FastQC and aligned to the A. nidulans FGSC_A4 genome (79, 80) using TopHat (v2.0.6) (81) default settings, with exceptions (minimum intron length, 10; maximum intron length, 4,000; maximum alignments, 40; minimum read length, 20). Strand-specific reads were separated using SAMtools view (v1.1) (82). Strand-specific transcripts were identified using AspGD annotations (s10_m03_r15) and Cufflinks (v2.1.1.7) (83, 84) default settings, with exceptions (max intron length, 4,000; bias correction, yes; multiread correction, yes). Identified transcripts from all growth conditions were combined into a single annotation using Cuffmerge guided by the reference annotation. Differential expression was determined using CuffDiff (84) and cummeRbund (v2.8.2) (85).

### Bioinformatics and *in silico* analyses.

DNA and protein sequences were downloaded from the Aspergillus Genome Database, AspGD (www.aspgd.org [83]), the *Saccharomyces* Genome Database, SGD (www.yeastgenome.org [86]), the Broad Institute genomes database (www.broadinstitute.org), the NCBI protein database (www.ncbi.nlm.nih.gov/protein/), and the EMBL-EBI Pfam database (http://pfam.xfam.org [87]). Protein sequence database searches used BLAST (https://blast.ncbi.nlm.nih.gov/Blast.cgi). Protein conserved domains were identified using the NCBI Conserved Domain Database (88). Pairwise protein sequence comparisons, and percent identity and similarity were calculated using EMBOSS Needle (EMBL-EBI) with default parameters. Sequences were analyzed in Geneious version 5.3.5, created by Biomatters (www.geneious.com). Multiple sequence alignments were made using ClustalW2 (89) or Clustal Omega (90) on the EMBL-EBI server (http://www.ebi.ac.uk/Tools/msa) and shaded using online BoxShade 3.2 (K. Hofmann and M. D. Baron) at ExPASy (https://embnet.vital-it.ch/software/BOX_form.html). Predicted subcellular localization of proteins was determined using Predotar 1.03 (https://urgi.versailles.inra.fr/predotar/ [42]), TargetP v1.1 (http://www.cbs.dtu.dk/services/TargetP/ [40, 41]), and DeepLoc-1.0 (https://services.healthtech.dtu.dk/service.php?DeepLoc-1.0 [43]). Colinearity of syntenic regions was illustrated using the GBrowse genome browser of FungiDB with genomes clustered based on whole-genome phylogenies (91–93, 99).

### Phylogenetic analyses.

The Pfam database (http://pfam.xfam.org/ [87]) was used to identify orthologs in the isocitrate/isopropylmalate dehydrogenase family (PF00180) and the aminotransferase class IV family (PF01063). Protein sequences were aligned and phylogenies were constructed using MAFFT (94). The neighbor-joining method with 100 bootstraps was used to generate consensus unrooted phylogenetic trees of β-IPM dehydrogenases and BATs. Tree visualization and label editing used Interactive Tree Of Life (iTOL) (95).

### Data availability.

RNA-seq fastq files and bigwigs have been deposited in NCBI's Gene Expression Omnibus (96) and are accessible through GEO Series accession number (GSE145035).
