# Supplementary material for: Duplication and Functional Divergence of Branched-Chain Amino Acid Biosynthesis Genes in Aspergillus nidulans
Source: mBio. 2021 Jun 22;12(3):e00768-21. doi: 10.1128/mBio.00768-21 (PMC8262921; doi:10.1128/mBio.00768-21)
Supplement: FIG S1 [file mbio.00768-21-sf001.pdf]

```
A. nig Leu2B    1  MSGTRAYNILVLPGDGIGPEVMAEAIKVLRTFNS..SSMQFHLQEELIGGISIDTHGHSV
A. nid AN2793   1  ~MSEKSYNILVLPGDGIGPEVMAEATKILSLFNT..STVRFRTQTELIGGCSIDTHGKSV
S. cer Leu2p    1  ~~MSAPKKIVVLPGDHVGQEITAEAIKVLKAISDVRSNVKFDFENHLIGGAAIDATGVPL
A. nig Leu2A    1  ~~~MPAYNIVVFAGDHWGPEVTAEAIKVLRVIEKSRDDITLNLQDHLLGGASIDATANPL
A. nid AN0912   1  ~~~MPSYNIVVFGGDHCGPEVTAEAVKILRVIEKSRDDITFNLQDHLLGGCSIDATGSPL

                                                 Substrate binding loop
A. nig Leu2B   59  TQPVKDAAVAADAVLFAAVGGSKVDHIRRGLDGPEGGLLQVRKAMDIYANLRPCSVDVPS
A. nid AN2793  58  TQAVLDAAVSSDAVLFAAVGGPKWDHIRRGLDGPEGGLLQVRKAMDIYANLRPCSVDSPS
S. cer Leu2p   59  PDEALEASKKADAVLLGAVGGPKWGT...GSVRPEQGLLKIRKELQLYANLRPCNFASDS
A. nig Leu2A   58  TDEALAAAKNADAVLLGAIGGPKWGT...GAVRPEQGLLNVRKEMGTFGNLRPCNFAAPS
A. nid AN0912  58  TDQALEAAKNADAVFLGAIGGPEWGT...GAVRPEQGILKLRKEMGTFANLRPCNFAAPS
                                                   E[QKR]XLLXXR

A. nig Leu2B  119  REIARDFSPFRQEVIEGVDFVVVRENCGGAYFGKKVEEE..NYAMDEWGYSTTEIQRIAR
A. nid AN2793 118  REIARDFSPFRQDVIEGVDFVVVRENCGGAYFGKKVEED..DYAMDEWGYSASEIQRITR
S. cer Leu2p  116  L...LDLSPIKPQFAKGTDFVVVRELVGGIYFGKRKEDDGDGVAWDSEQYTVPEVQRITR
A. nig Leu2A  115  L...VEHSPLKASVCEGVDFNIIRELTGGIYFGDRKKMTA.AATMDTEPYSRAEIERITP
A. nid AN0912 115  L...VESSPLRPEICRGVDFNIIRELTGGIYFGERKEDDGSGFALDTEPYSRAEIERITR

A. nig Leu2B  177  LAAELALRHDPPWPVISLDKANVLASSRLWRRVVENTISVEYPQVKLVHQLADSASLIMA
A. nid AN2793 176  LSAELALRHDPPWPVISLDKANVLASSRLWRRVVEKTMSEEYPQVKLVHQLADSASLIMA
S. cer Leu2p  173  MAAFMALQHEPPLPIWSLDKANVLASSRLWRKTVEETIKNEFPTLKVQHQLIDSAAMILV
A. nig Leu2A  171  .RAHLALQHNPPLPVWSLDKANVLATSRLWRKTVTEIMAKEFPQLKIEHQLIDSAAMIMV
A. nid AN0912 172  LGAHLALQHNPPLPVWSLDKANVLATSRLWRKTVTEIMAKEFPQLKLEHQLIDSAAMIMV

A. nig Leu2B  237  TDPRVLNGVILADNTFGDMLSDQAGSLIGTLGVLPSASLDGLPHPGKQEKVRGLYEPTHG
A. nid AN2793 236  TNPRALNGVILADNTFGDMVSDQAGSLVGTLGVLPSASLDGLPKPGEQRKVHGLYEPTHG
S. cer Leu2p  233  KNPTHLNGIIITSNMFGDIISDEASVIPGSLGLLPSASLASLPD..KN.TAFGLYEPCHG
A. nig Leu2A  230  KNPRQLNGIIVTSNLFGDIISDEASVIPGSLGLLPSASLSGIPD..GKGRVNGIYEPIHG
A. nid AN0912 232  KDPRKLNGIVITSNLFGDIISDEASVIPGSLGLLPSASLSSIPD..GKGKVNGIYEPIHG
                   NAD binding
A. nig Leu2B  297  SAPTIAGKNIANPTAMILCVSLMFRYSFNMENEARQIEDAVRAVLDRGLRTPDLGGNSST
A. nid AN2793 296  SAPTIAGKNIANPTAMILCVALMFRYSFNMEAEARQIEAAVRTVLDKGIRTSDLGGSTGT
S. cer Leu2p  290  SAPDLPK.NKVNPIATILSAAMMLKLSLNLPEEGKAIEDAVKKVLDAGIRTGDLGGSNST
A. nig Leu2A  288  SAPDIAGKGIVNPVAAILSVAMMMQYSFGRFDEARAIEAAVRNVLESGVRTGDIGGKATT
A. nid AN0912 290  SAPDISGKGIVNPVAAILSVGLMMQYSFALFEEARAVATAVSNVIEAGVRTGDIGGKAST
                 HGSAPDI
A. nig Leu2B  357  QEFGDAVVAALQGKY~~
A. nid AN2793 356  REFGDAVVAALKGEL~~
S. cer Leu2p  349  TEVGDAVAEEVKKILA*
A. nig Leu2A  348  SEVGDAVAAELEKLLK~
A. nid AN0912 350  KEVGDAVAAELEKLLKK
```

Figure S1. Clustal Omega alignment of β-isopropylmalate dehydrogenases

Clustal Omega alignment of Leu2p from *S. cerevisiae* (S. cer) with, AN0912 (LeuD) and AN2793 (LeuE) from *A. nidulans* (A. nid), and Leu2A and Leu2B from *A. niger* (A. nig). The substrate-binding loop (green) and the NAD-binding motif (blue). Shading was performed with Boxshade with a minimum of 0.6 identity (black) or similarity (gray).
