# Supplementary material for: Duplication and Functional Divergence of Branched-Chain Amino Acid Biosynthesis Genes in Aspergillus nidulans
Source: mBio. 2021 Jun 22;12(3):e00768-21. doi: 10.1128/mBio.00768-21 (PMC8262921; doi:10.1128/mBio.00768-21)
Supplement: FIG S2 [file mbio.00768-21-sf002.pdf]

Figure S2A AN0912 (*leuD*)

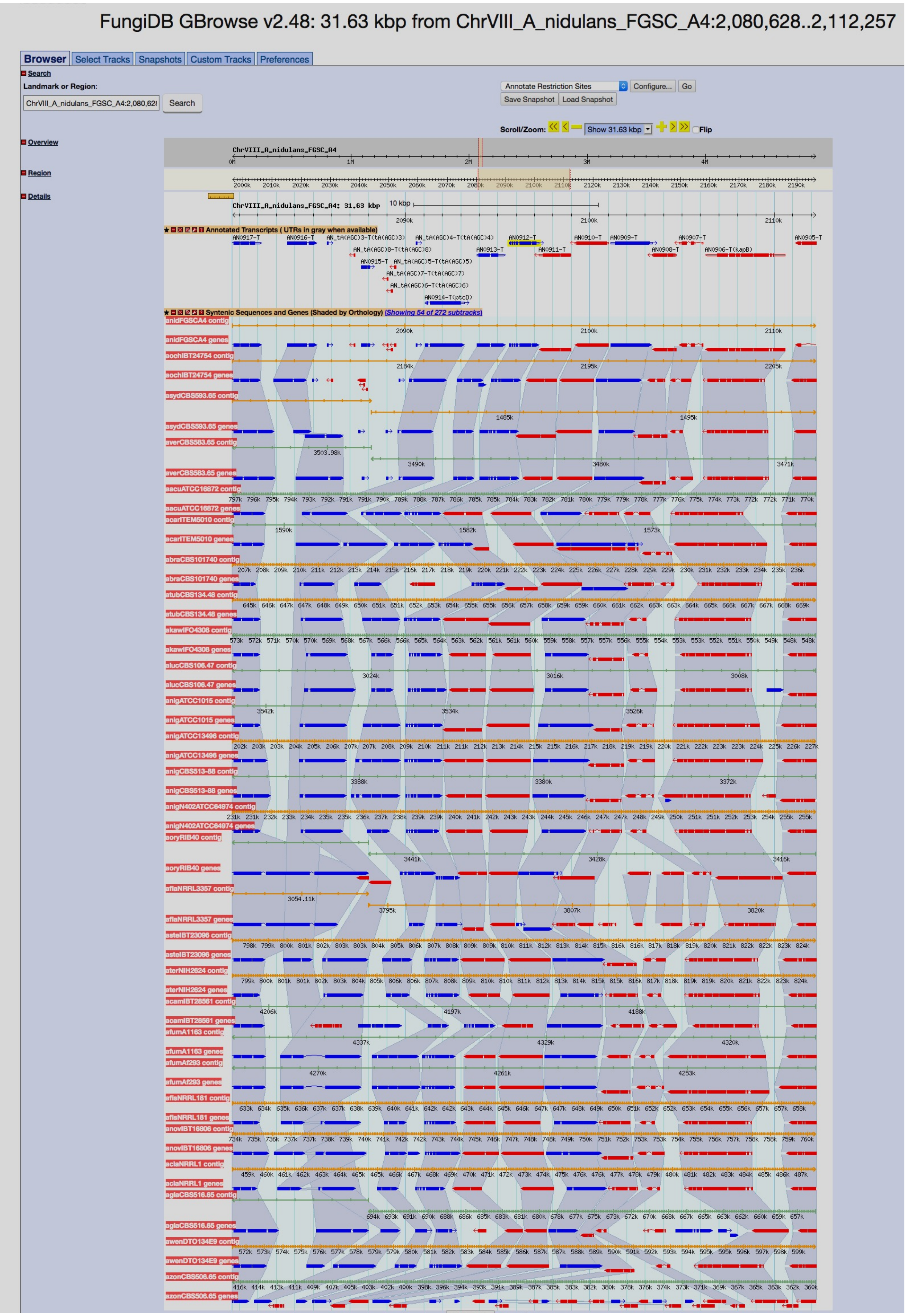

Figure S2B   AN2793 (*leuE*)

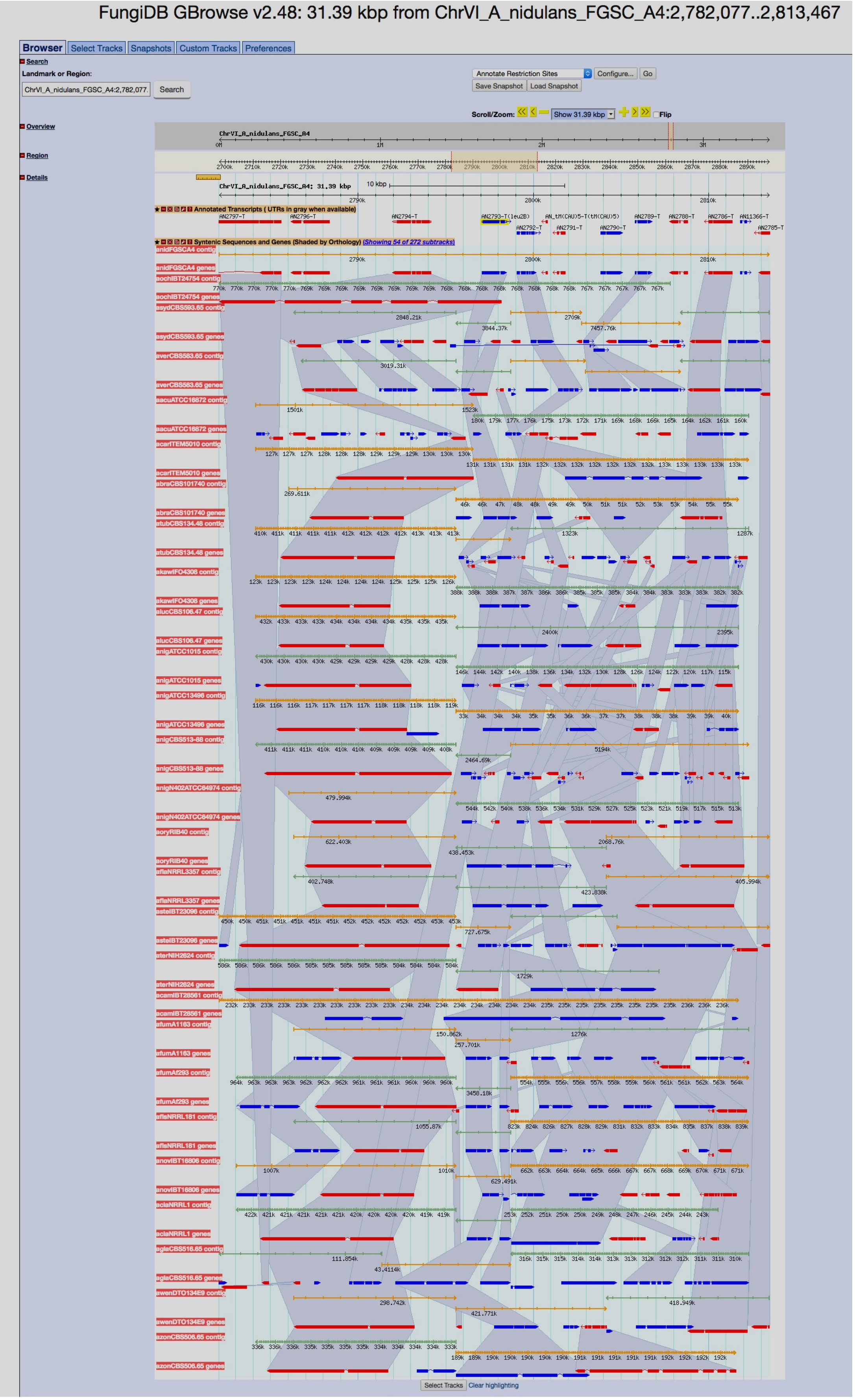

Figure S2. Colinearity of β-isopropylmalate dehydrogenase genes in Aspergilli.

The colinearity of syntenic regions for (A) AN0912 (*leuD*) and (B) AN2793 (*leuE*) was illustrated using the GBrowse genome browser of FungiDB with genomes displayed in the order: *Aspergillus nidulans* FGSC A4, *Aspergillus ochraceoroseus* IBT 24754, *Aspergillus sydowii* CBS 593.65, *Aspergillus versicolor* CBS 583.65, *Aspergillus aculeatus* ATCC 16872, *Aspergillus carbonarius* ITEM 5010, *Aspergillus brasiliensis* CBS 101740, *Aspergillus tubingensis* CBS 134.48, *Aspergillus kawachii* IFO 4308, *Aspergillus luchuensis* CBS 106.47, *Aspergillus niger* ATCC 1015, *Aspergillus niger* ATCC 13496, *Aspergillus niger* CBS 513.88, *Aspergillus niger* strain N402 (ATCC64974), *Aspergillus oryzae* RIB40, *Aspergillus flavus* NRRL3357, *Aspergillus steynii* IBT 23096, *Aspergillus terreus* NIH2624, *Aspergillus campestris* IBT 28561, *Aspergillus fumigatus* A1163, *Aspergillus fumigatus* Af293, *Aspergillus fischeri* NRRL 181, *Aspergillus novofumigatus* IBT 16806, *Aspergillus clavatus* NRRL 1, *Aspergillus glaucus* CBS 516.65, *Aspergillus wentii* DTO 134E9, *Aspergillus zonatus* CBS 506.65.
