# Supplementary material for: Duplication and Functional Divergence of Branched-Chain Amino Acid Biosynthesis Genes in Aspergillus nidulans
Source: mBio. 2021 Jun 22;12(3):e00768-21. doi: 10.1128/mBio.00768-21 (PMC8262921; doi:10.1128/mBio.00768-21)
Supplement: FIG S3 [file mbio.00768-21-sf003.pdf]

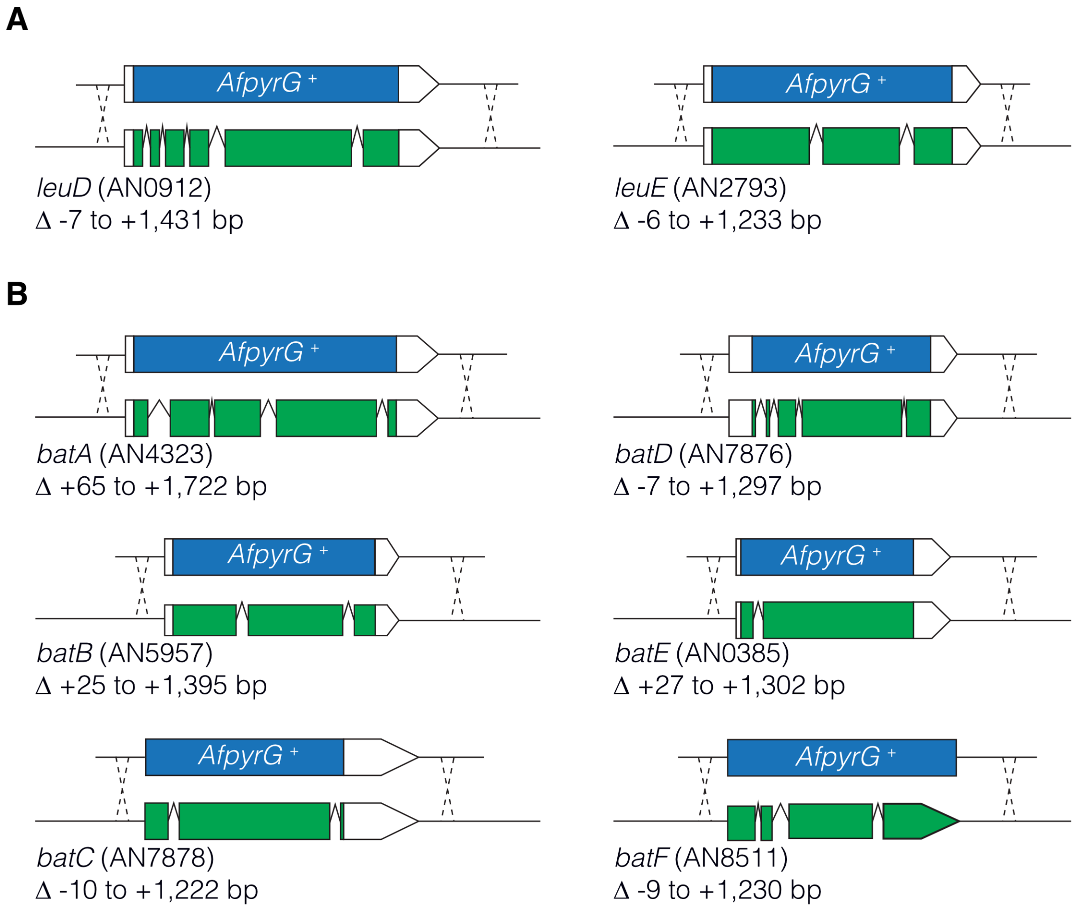

Figure S3. Gene knockout cassettes.

Strategy for the deletion two candidate β-IPM dehydrogenase-encoding genes, *leuD* and *leuE,* **(A)** and six candidate BCAA aminotransferase-encoding genes, *batA*, *batB*, *batC*, *batD*, *batE*, and *batF*, **(B)** using cassettes that gene replaced the coding region with the *A. fumigatus pyrG* selectable marker (*AfpyrG*$^{+}$).
