# Supplementary material for: Duplication and Functional Divergence of Branched-Chain Amino Acid Biosynthesis Genes in Aspergillus nidulans
Source: mBio. 2021 Jun 22;12(3):e00768-21. doi: 10.1128/mBio.00768-21 (PMC8262921; doi:10.1128/mBio.00768-21)
Supplement: FIG S4 [file mbio.00768-21-sf004.pdf]

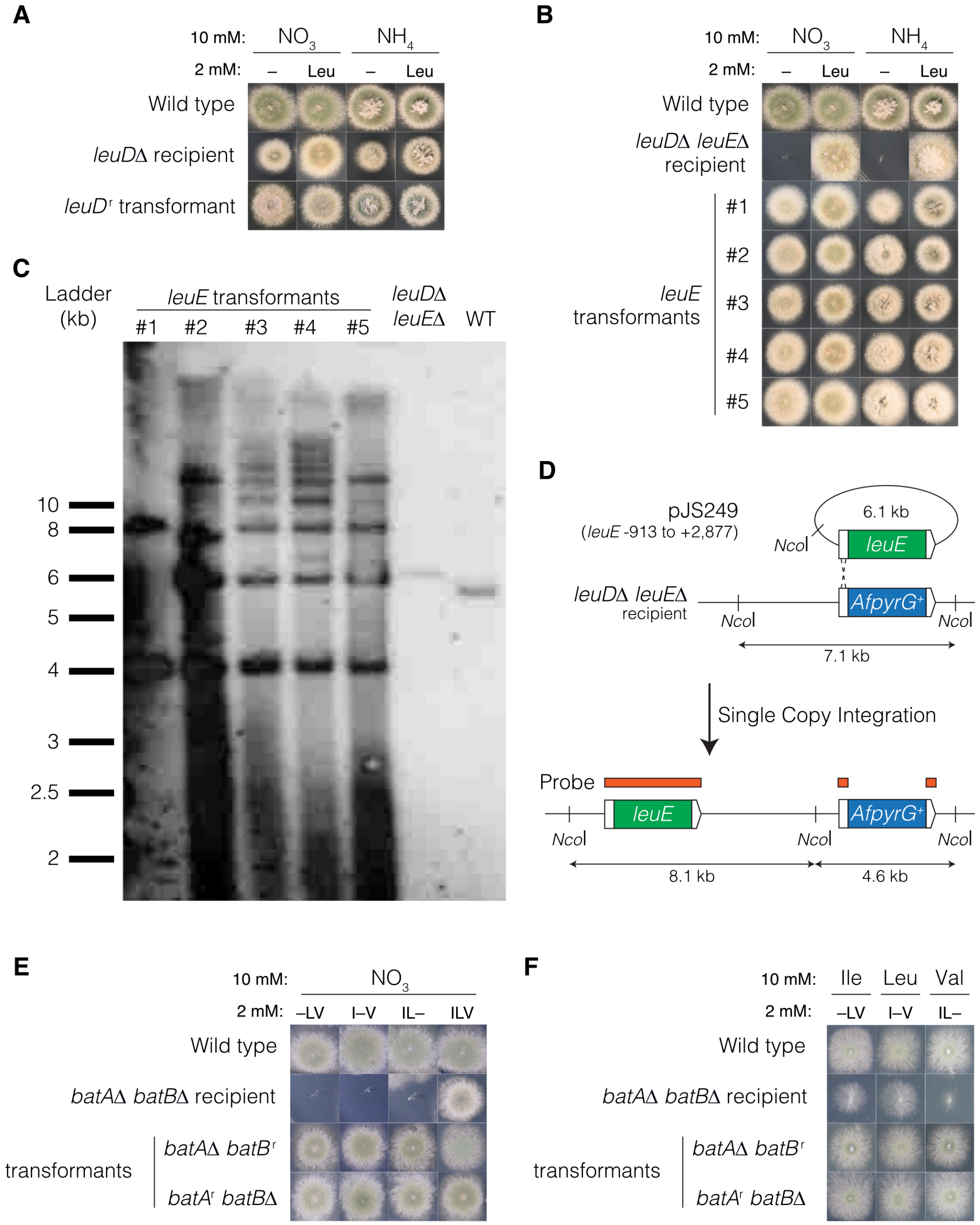

Figure S4: Complementation analysis of deleted genes.

A) Transformants of the *leuD*Δ mutant (RT411) with PCR-amplified wild type *leuD* DNA were grown at 37ºC for 2 d on solid supplemented ANM with (Leu) or without (–) 2 mM leucine, and with 10 mM ammonium (NH_4) or nitrate (NO_3) as the nitrogen source. *leuD*^{r}

indicates the reconstructed wild type *leuD* by gene replacement of *leuD*Δ*::AfpyrG.* **B)** Wild type (MH1), *leuD*Δ *leuE*Δ (RT444), and transformants of RT444 with a plasmid carrying wild type *leuE* were grown at 37ºC for 2 d on solid supplemented ANM with (Leu) or without (–) 2 mM leucine, and with 10 mM ammonium (NH_4) or nitrate (NO_3) as the nitrogen source. Transformant #1 is a single copy *leuE* integrant by homology via the *leuE*Δ flanking sequence, while transformants #2-#5 are multicopy transformants. **C)** Southern analysis of *Nco*I-digested genomic DNA from *leuE* transformants, the *leuD*Δ *leuE*Δ recipient, and wild type, hybridized with the *leuE* plasmid insert as probe. **D)** Single copy homologous integration of the *leuE* plasmid into the *leuD*Δ *leuE*Δ recipient strain. Only integration via crossover at the left flank of *leuE* is shown. *Nco*I restriction sites and restriction fragment lengths are indicated. Sequences bound by the *leuE* probe are represented by the orange boxes. **E)** Wild type (MH1), *batA*Δ *batB*Δ (RT457) and transformants of RT457 with PCR-amplified wild type *batA* (*batA*^{r} *batB*Δ) or *batB* (*batA*Δ *batB*^{r}) DNA, grown under ILV anabolic conditions at 37ºC for 2 days on solid supplemented ANM with 10 mM nitrate (NO_3) as the nitrogen source and combinations of 2 mM each of isoleucine (I), leucine (L), and valine (V) to supplement potential auxotrophies. *batA*^{r} and *batB*^{r} indicate the reconstructed wild type allele. **F)** Strains from panel E grown under ILV catabolic conditions at 37ºC for 2 days on solid supplemented ANM with 10 mM of either Isoleucine (Ile), Leucine (Leu), or Valine (Val) as the predominant nitrogen source and combinations of 2 mM each of isoleucine (I), leucine (L), and valine (V) to supplement potential auxotrophies. In **E** and **F**, – represents an omitted amino acid.
