# Supplementary material for: Duplication and Functional Divergence of Branched-Chain Amino Acid Biosynthesis Genes in Aspergillus nidulans
Source: mBio. 2021 Jun 22;12(3):e00768-21. doi: 10.1128/mBio.00768-21 (PMC8262921; doi:10.1128/mBio.00768-21)
Supplement: FIG S5 [file mbio.00768-21-sf005.pdf]

```
Sc_Bat1p      1 ~~~~~~~~~~~~~~~~~~~~~~~~~~~~~~~~~~~~~~~~~~~~~~~~~~~~~~~~~~~~~~~~~~~MLQ
Sc_Bat2p      1 ~~~~~~~~~~~~~~~~~~~~~~~~~~~~~~~~~~~~~~~~~~~~~~~~~~~~~~~~~~~~~~~~~~~~~~
AN4323        1 MRFGCGFLRSLNCKLRLVTQEAPFNLYHDLRYPSAILLATGATADMKSLHQLARPRALPSFAAPRAQSAR
AN5957        1 ~~~~~~~~~~~~~~~~~~~~~~~~~~~~~~~~~~~~~~~~~~~~~~~~~~~~~~~~~~~~~~~~~~~~~~
AN7878        1 ~~~~~~~~~~~~~~~~~~~~~~~~~~~~~~~~~~~~~~~~~~~~~~~~~~~~~~~~~~~~~~~~~~~MAR
AN7876        1 ~~~~~~~~~~~~~~~~~~~~~~~~~~~~~~~~~~~~~~~~~~~~~~~~~~~~~~~~~~~~~~~~~~~~~~
AN0385        1 ~~~~~~~~~~~~~~~~~~~~~~~~~~~~~~~~~~~~~~~~~~~~~~~~~~~~~~~~~~~~~~~~~~~~~~
AN8511        1 ~~~~~~~~~~~~~~~~~~~~~~~~~~~~~~~~~~~~~~~~~~~~~~~~~~~~~~~~~~~~~~~~~~~~~~

Sc_Bat1p      4 RHSLKL.GKFSIRTLATGAPLDASKLKITRNPNPS..KPRPNEELV..........FGQTF...TDHMLT
Sc_Bat2p      1 ~~~~~~~~~~~~~~~MTLAPLDASKVKITTTQHAS..KPKPNSELV..........FGKSF...TDHMLT
AN4323       71 SNRLWQRCFSATRAASEGAQLDPSKLTITKTSTPK..ELLPAKDLV..........FGKNF...TDHMFT
AN5957        1 ~~~~~~~~~~MGSIGNTLAELDASVVKITRSTELRHVPLPGSLEEL..........SHSYC...TDHMVT
AN7878        4 LLNRWHLPALVRRH....YSSRFQSLRIEKTQSPK..PLPDSTELQ..........FGRSF...TDHILK
AN7876        1 ~~~~~~~~~~~~~~~~~~~~~~~~~~~~~~~~~MSTFPAEPRSDIEWE.......KVGVSFLDVNGHVES
AN0385        1 ~~~~~~~~~~~~~~~~~~~~~~~~~~~~~~~MASQSFPPPPVDTIDWS.......NIGFKVREVNGHVES
AN8511        1 ~~~~~~~~~~~~~~~~~~~~~~~~~~~~~~~~~~~~~~~~~~~MTEWKVTIIHADLTSASLTPANGDVQC

Sc_Bat1p     58 IPWSAKEGWGTPHIKPYGNLSLDPSACVFHYAFELFEGLKAYRTPQN.TITMFRPDKNMARMNKSAARIC
Sc_Bat2p     41 AEWTAEKGWGTPEIKPYQNLSLDPSAVVFHYAFELFEGMKAYRTVDN.KITMFRPDMNMKRMNKSAQRIC
AN4323      126 VEWTAKDGWLAPQIVPYQKLQLDPSACVFHYAFECFEGMKAYKDSKG.QIRLFRPDKNMQRLNKSSARIA
AN5957       48 ARWTAAGGWETPEVKPFQNLSIPPTASCLHYATECFEGMKVYRGFDG.KLRLFRPDLNGERLSNSAVRAS
AN7878       55 LEWTTTQGWSDAQITPYDNLRLDPASCVLHYAFTCFEGMKAYKDPHG.NARLFRPEENLARLNRSAARLA
AN7876       31 DFNYQTGTWSEPVFVQDHYLKVHGLAPGLNYGQQVFEGMKAYRDPNG.QIQIFRPTDHALRMQRSCDAVS
AN0385       33 HYTPATKSWSPPKLVKSPYLPIHGMAPGLNYGQQAYEGLKAFRHPNNSKITIFRPDRNALRMQRSASFIS
AN8511       28 QFSRKFGKWSAPLFIEDPFLRVHGLAPVFNYGQEVYEGLKGIPGTESQWPTP.HPPLH............

Sc_Bat1p    127 LPTFESEELIKLTGKLIEQDK..HLVPQGNGYSLYIRPTMIGTSKGLGVGTPSEALLYVITSPVGPYYKT
Sc_Bat2p    110 LPTFDPEELITLIGKLIQQDK..CLVPEGKGYSLYIRPTLIGTTAGLGVSTPDRALLYVICCPVGPYYKT
AN4323      195 LPTVDGEALTKLIGELVKLDS..RFIPDARGYSLYLRPTMIGTQSTLGVGPPGSALLFVIASPVGPYYPT
AN5957      117 LPSFRFQELKTLIAKLMQIDGLRWLPKDQPGRFLYLRPTLIGSGTQLGVQAPAEALLFIIAVPWPDPA.T
AN7878      124 LPTFEESGVLEFLAKYVDLEK..RFIPHLPGHSLYLRPTLLGTDASISVSRPRSALLFVIASPMGDYFAN
AN7876      100 IPSIPESLFWASVNLAVAKN.SEFVPPHASEAAMYIRPLAFGSGGWMPVAAGPQYKFVVYALPFCAYHGT
AN0385      103 IPPVPEDLFLEAVELAVGAN.AGFVPPHETGAAMYIRPLIFGSSAQLGLSPPEEYTFVVFVMPTGVYHGV
AN8511       85 .SPGSRTPLPRSVNLAVAQN.AEFVPSHESQGMLYIRPILFGSSACIQLTPPDKYTFCVYVTPVAAYNGI

                                                *
Sc_Bat1p    195 GFKA........VRLEATDYATRAWPGGVGDKKLGANYAPCILPQLQAAKRGYQQNLWLFGP....EKNI
Sc_Bat2p    178 GFKA........VRLEATDYATRAWPGGCGDKKLGANYAPCVLPQLQAASRGYQQNLWLFGP....NNNI
AN4323      263 GFKA........ISLEATDYAVRAWPGGVGDKKLGANYAPCIVPQLEAASRGFQQNLWLFGE....EEYV
AN5957      186 RLKATPGEALGLKLLTSAPDTIRAWPGGFGYAKLGANYGPSLAAHGKAQAQGFDQVLWLFGE....DRQV
AN7878      192 GMKA........VTLQATRSPVRAWPGGVGEFKVGGNYAPSIVPQEEAAEAGSQQNLWLLADRESGEEFV
AN7876      169 ..LP.....VDAVVL...EELDRAAPLGVGNVKVGGNYAPVLKWSDKARKEGFGITLHLDSKT...RGEI
AN0385      172 ..HA.....VDALIL...EDFDRAAPEGTGSAKVGGNYAPVLRHSAKAHAEGFGITLHLDSRT...RSEI
AN8511      153 ..NP.....LDALIL...EGFDRAAPRGTGSGKVGGNYAPVMKWSDQARREGYAITLHLDSAT...RSEV
                                                *

Sc_Bat1p    253 TEVGTMNVFFVFLNKVTGKKELVTAPLDG.TILEGVTRDSVLTLARDKLDP.....QEWDINERYYTITE
Sc_Bat2p    236 TEVGTMNAFFVFKDSKTGKKELVTAPLDG.TILEGVTRDSILNLAKERLEP.....SEWTISERYFTIGE
AN4323      321 TEVGTMNLFIALKNKETGKKELVTANLDG.TILEGVTRDSVLALARERLVP.....KGWQVSERKIRMAE
AN5957      252 TEAGASNFFIVWENAQTGKRELVTAPLENQLILPGVTRRSVLELARSRLNQAVGDLEAVEVVEKTFTIWD
AN7878      254 TEAGTMNLFVVWVSSSTGKKELVTPPLDG.TILPGVTRMSILELARERLEG...DRSGIEVVERRITMRE
AN7876      226 DEFSTSGFVGIKYTESGGEKGYTLVVPNSQCIIKSVTSTSVVEVAR.S........LGWRVEVRPIPYDE
AN0385      229 DEFSTSGMIAVKKNKESGK..VTLVQPDSPNVIDSVTAASVCEIGKLW........FGYDVEKRRIPYEE
AN8511      210 EEFSTAGFVGVKEKADG..DGVAVVVPDSGNVVDSVTVRCILAVAA.R........MGWAVERRVIKYEE

Sc_Bat1p    317 VATRAKQGELLEAFGSGTAAVVSPIKEIGWNNEDIHVPLLPGEQ...CGALTKQVAQWIADIQYGRV..N
Sc_Bat2p    300 VTERSKNGELLEAFGSGTAAIVSPIKEIGWKGEQINIPLLPGEQ...TGPLAKEVAQWINGIQYGET..E
AN4323      385 VAEAADEGRLLEVFGAGTAAIVSPVRTISYRGRLVNCGLKETEE...AGEIASQMKNWIEGIQYGDE..E
AN5957      322 VEAAWKEGRVVEAFVCGTAFFITPVKLIRNGAVD..IQLLKPGQ...TAGYAAQIKSWLEAVMYGKDGAE
AN7878      320 LAAASKEGRLLEVFGAGTAVVVSPVRSIRWGDQCISCGLRDGEE...AGPMSLQMKTWLEEVQYGLV..E
AN7876      287 LEF......FDEVLAVGTAAMITSIRSITHRSKDQVFRYKTSDE...PGSACEKLSRHLKGIQKGDEKDT
AN0385      289 LNE......FDEVMAAGTAAALVPIRSITRRSSGNRFEYECGGEEAGGGEVCVKLLRTLKGIQLGKIEET
AN8511      269 LAS......FSEILACGTAVTVIPIKSITCKSRGNRFTYLEASV.SKPGPYAAKLAATLGDIQRGKVET~

Sc_Bat1p    382 YGNWSKTVADLN~~~~~~~~~~~~~~~~~~~~~~~~~~~~~~~
Sc_Bat2p    365 HGNWSRVVTDLN~~~~~~~~~~~~~~~~~~~~~~~~~~~~~~~
AN4323      450 H.PWSYVL~~~~~~~~~~~~~~~~~~~~~~~~~~~~~~~~~~~
AN5957      387 NHEWSYIIENESEK~~~~~~~~~~~~~~~~~~~~~~~~~~~~~
AN7878      385 H.PWSYRV~~~~~~~~~~~~~~~~~~~~~~~~~~~~~~~~~~~
AN7876      348 F.GWLKRVEEVTV~~~~~~~~~~~~~~~~~~~~~~~~~~~~~~
AN0385      353 L.GWNRVVKAPPAEWVGAADEKEEAGIEVPQACRMICTASNWY
AN8511      331 ~~~~~~~~~~~~~~~~~~~~~~~~~~~~~~~~~~~~~~~~~~~
```

Figure S5. Clustal Omega alignment of putative BCAA aminotransferases.

Figure S5. Clustal Omega alignment of putative BCAA aminotransferases.

Clustal Omega alignment of Bat1p and Bat2p from *S. cerevisiae* with AN4323 (BatA), AN5957 (BatB), AN7878 (BatC), AN7876 (BatD), AN0385 (BatE), and AN8511 (BatF) from *A. nidulans*. The co-factor binding residues (green), and mitochondrial-targeting signals (blue) are highlighted. The catalytic lysine is marked with a star above and below. Shading was performed with Boxshade with a minimum of 0.5 identity (black) or similarity (gray).
