# Supplementary material for: Duplication and Functional Divergence of Branched-Chain Amino Acid Biosynthesis Genes in Aspergillus nidulans
Source: mBio. 2021 Jun 22;12(3):e00768-21. doi: 10.1128/mBio.00768-21 (PMC8262921; doi:10.1128/mBio.00768-21)
Supplement: FIG S6 [file mbio.00768-21-sf006.pdf]

Figure S6A AN4323 (*batA*)

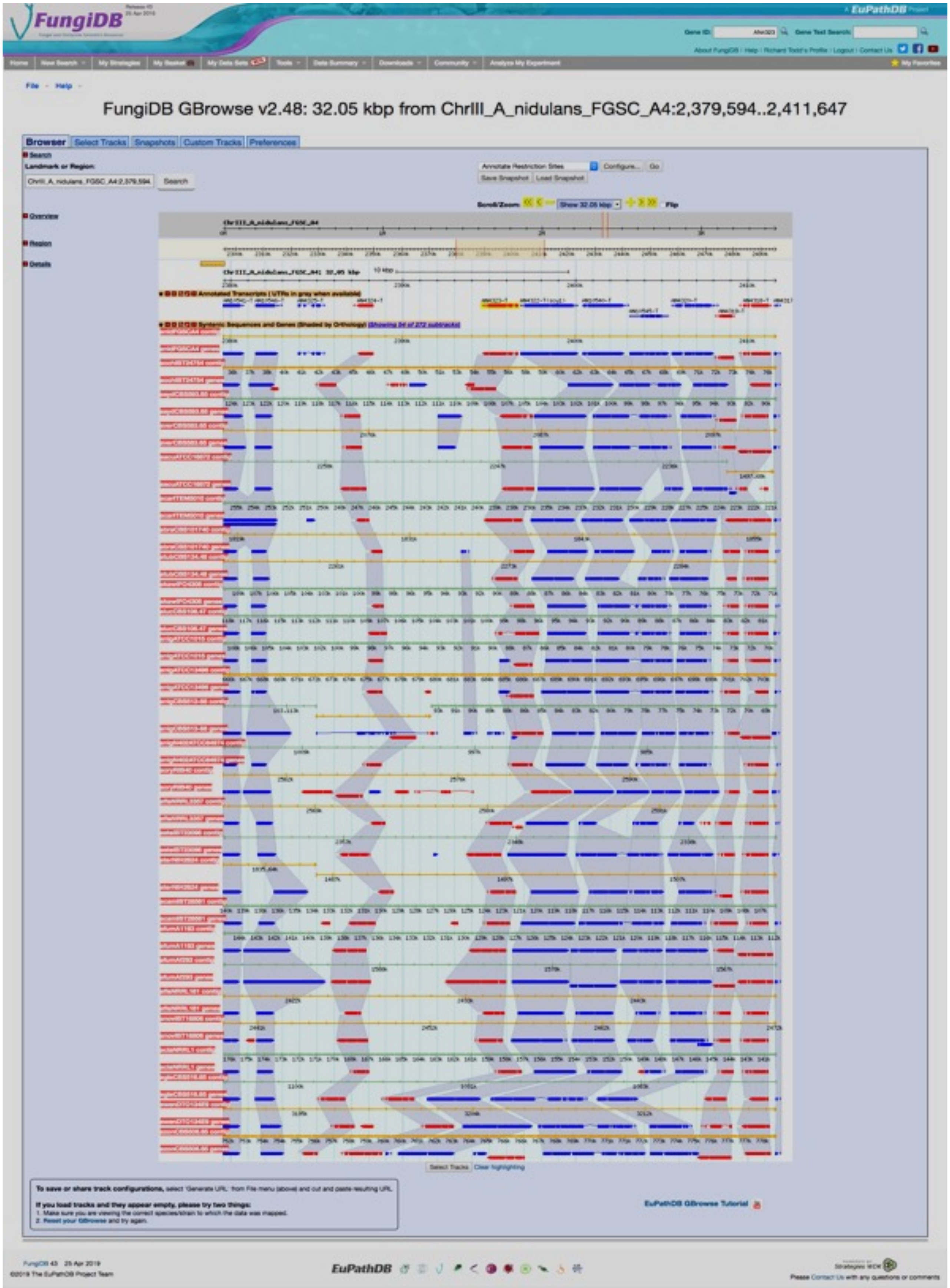

Figure S6B AN5957 (*batB*)

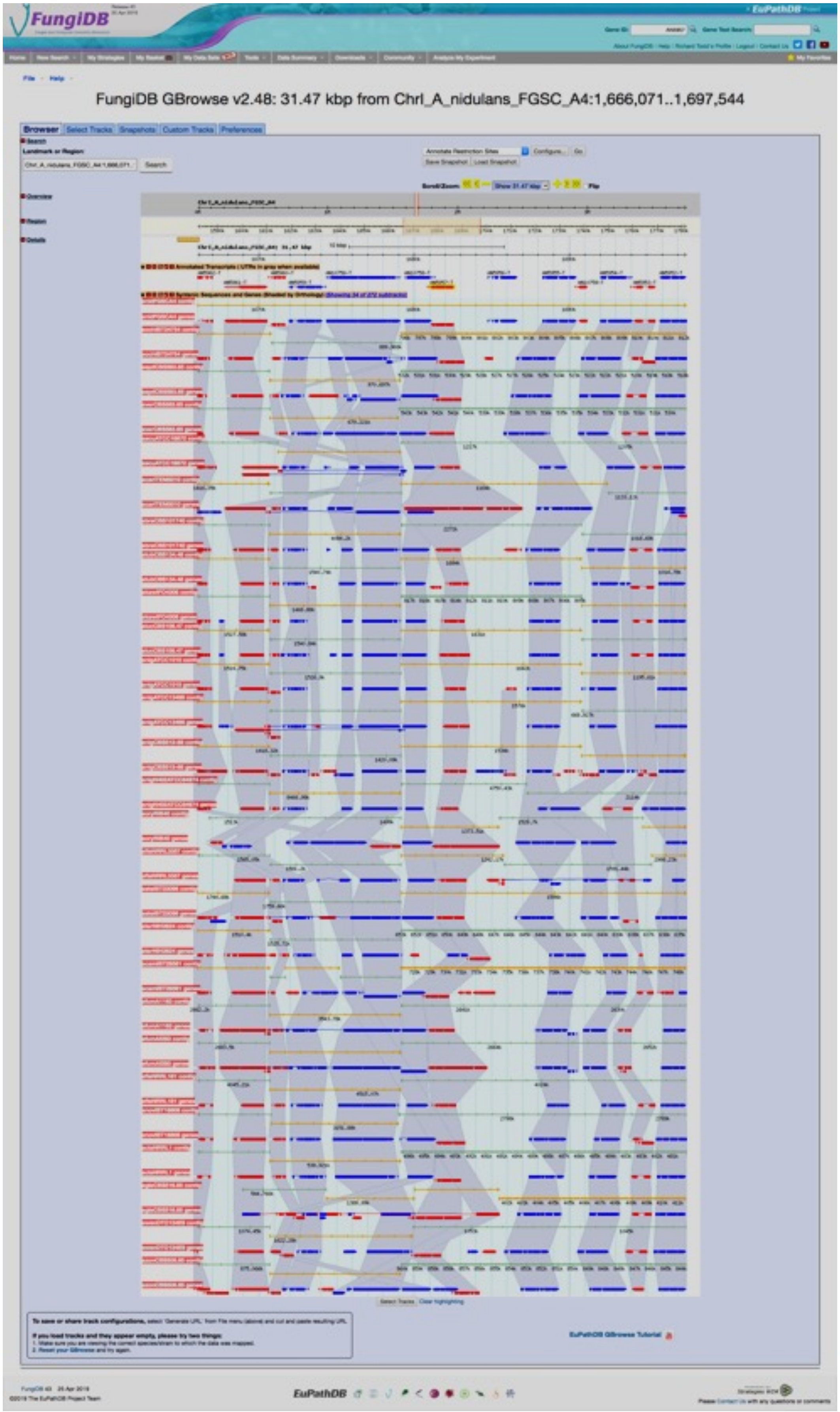

Figure S6C AN7878 (*batC*)

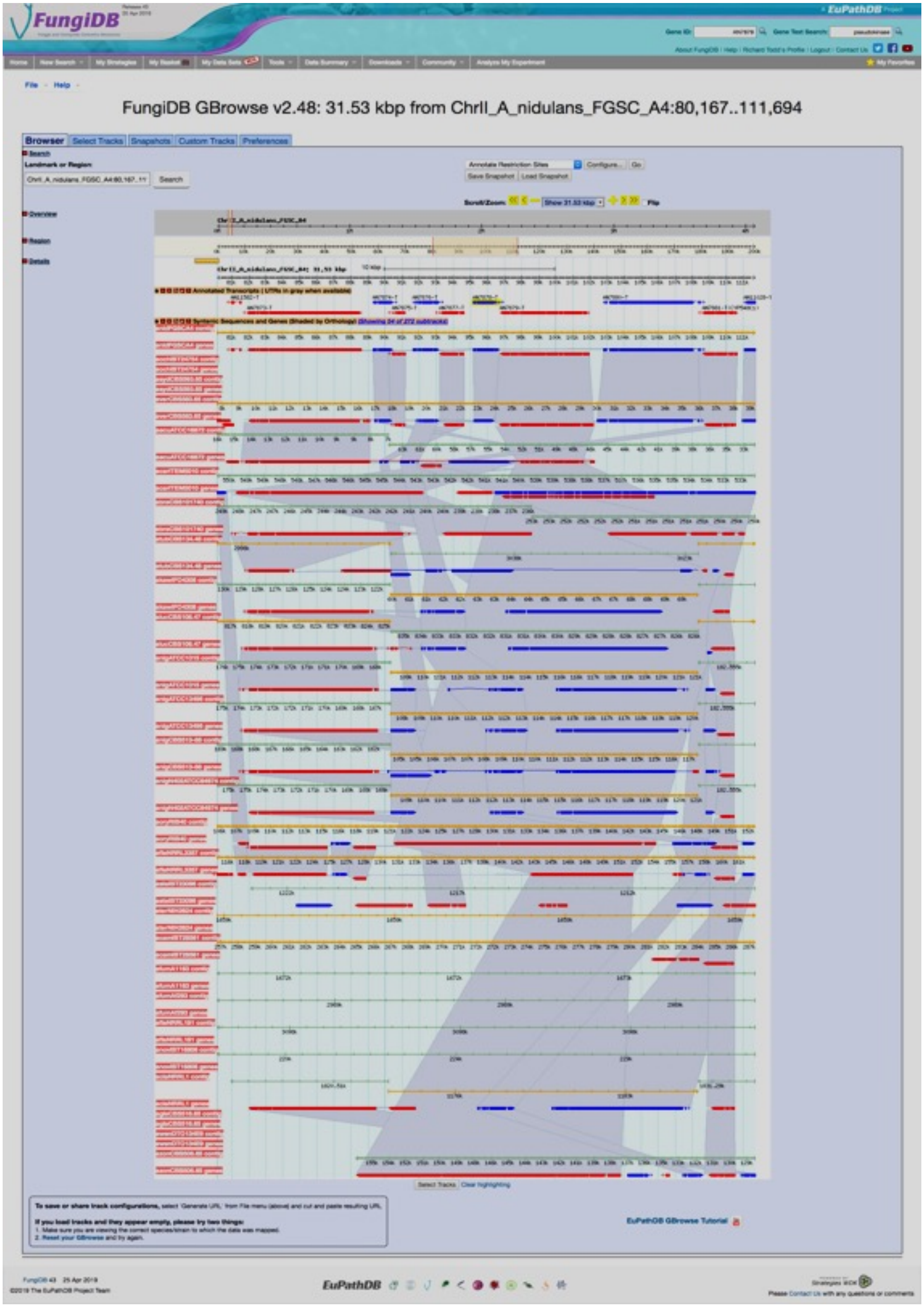

Figure S6D AN7876 (*batD*)

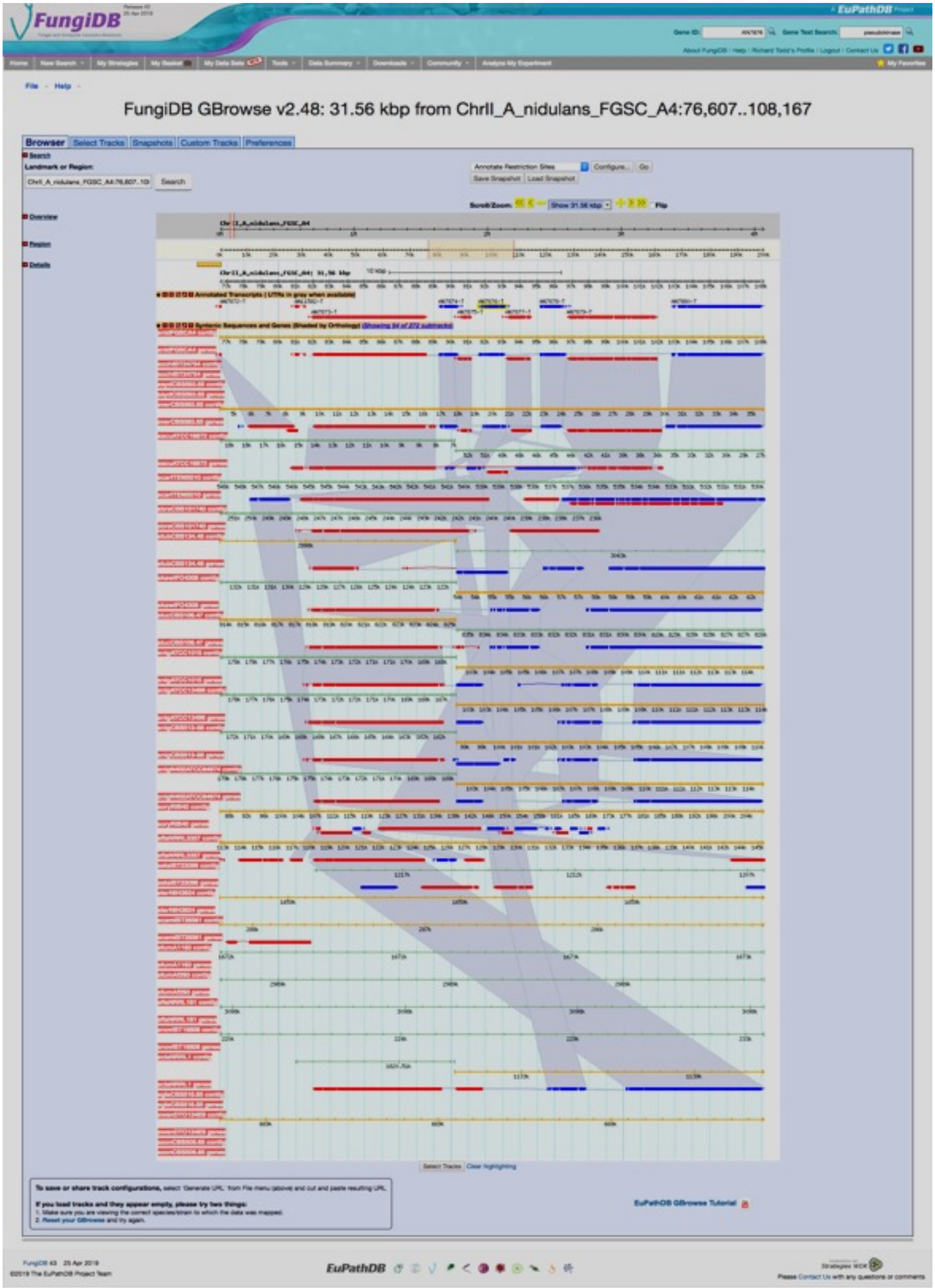

Figure S6E AN0385 (*batE*)

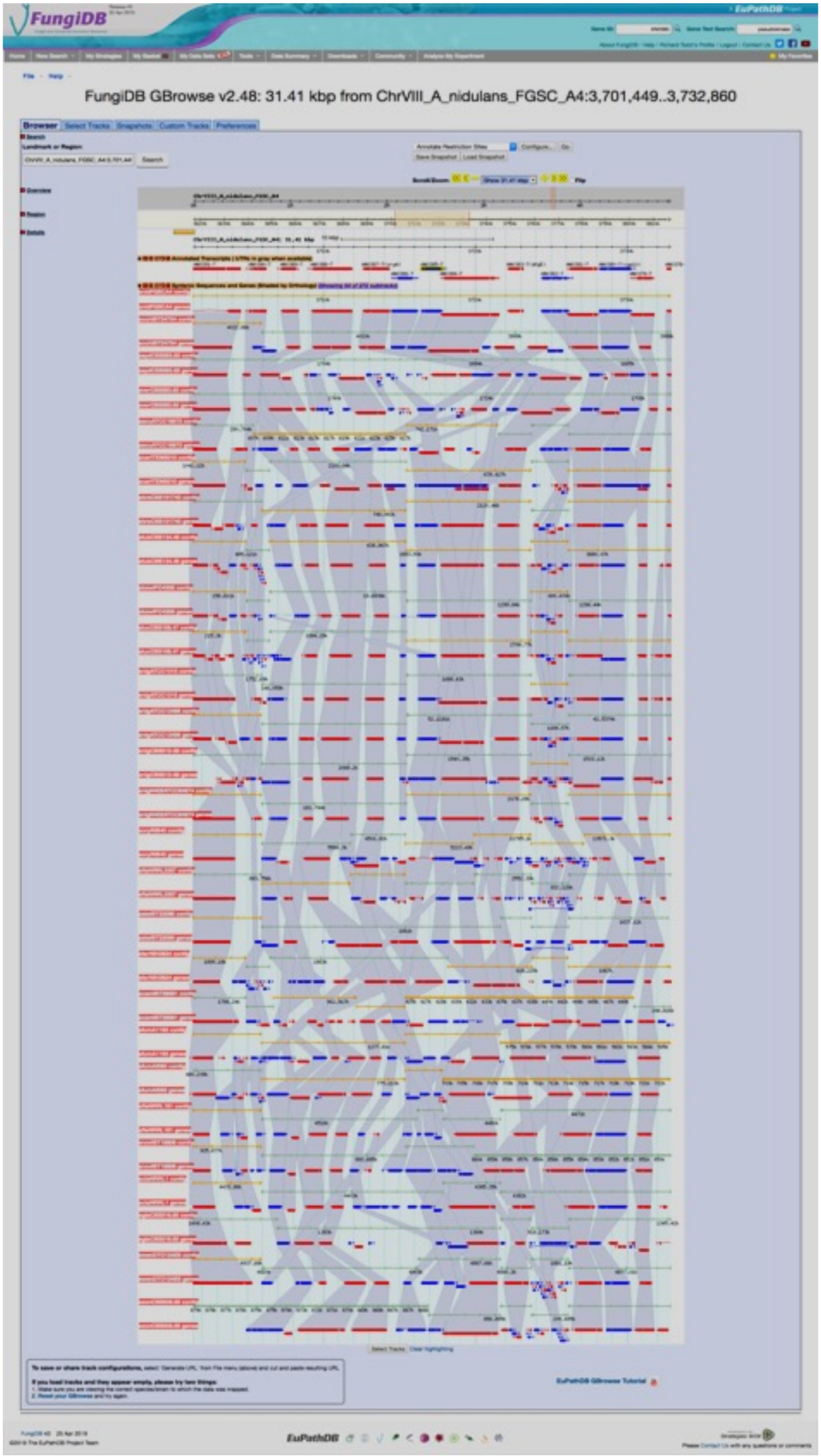

Figure S6F AN8511 (*batF*)

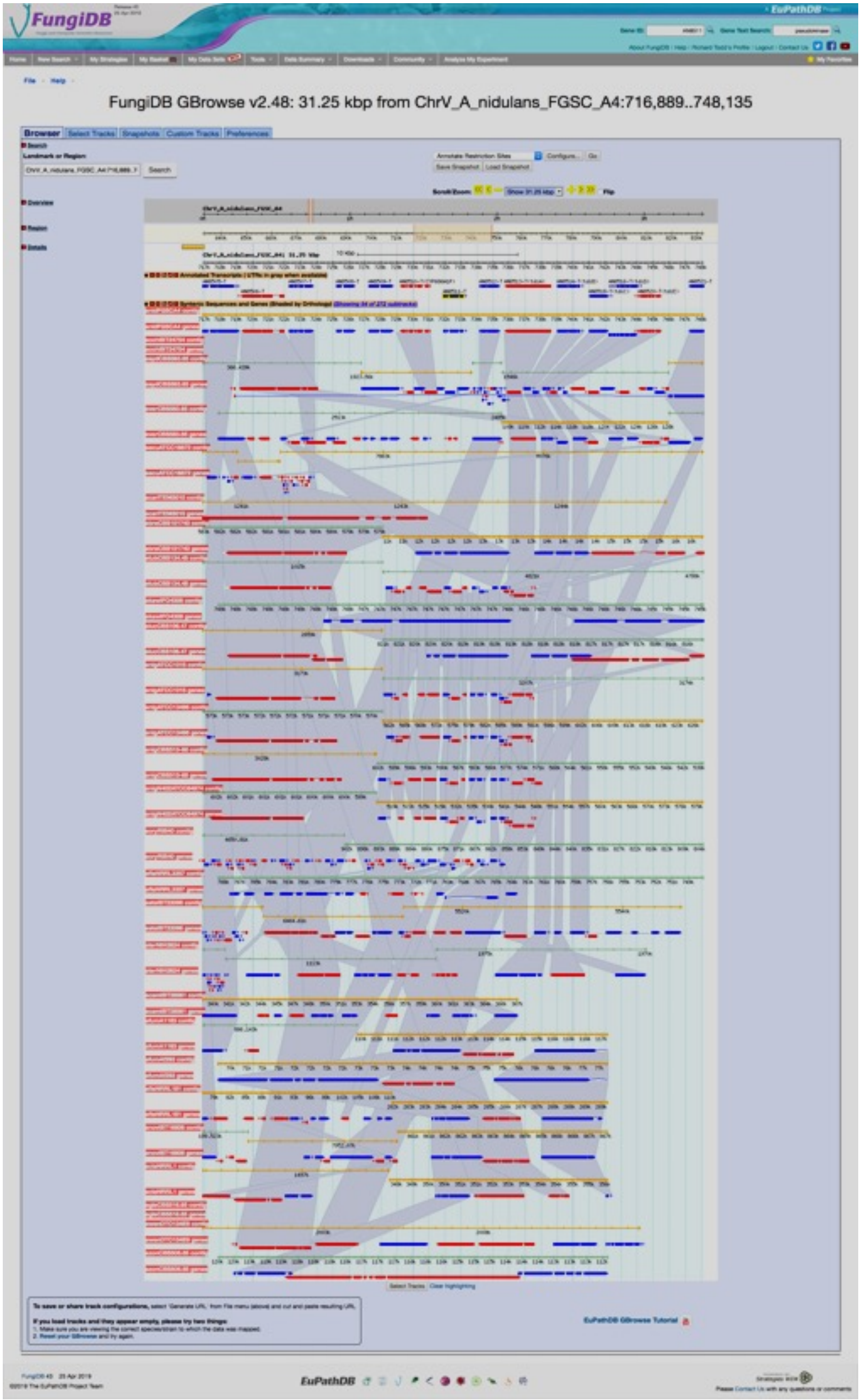

Figure S6. Colinearity of BCAA aminotransferase genes in Aspergilli.

The colinearity of syntenic regions for **(A)** AN4323 (*batA*), **(B)** AN5957 (*batB*), **(C)** AN7878 (*batC*), **(D)** AN7876 (*batD*), **(E)** AN0385 (*batE*), and **(F)** AN8511 (*batF*) was illustrated using the GBrowse genome browser of FungiDB with Aspergillus genomes displayed in the same order as in Figure S2.
