# Supplementary material for: Duplication and Functional Divergence of Branched-Chain Amino Acid Biosynthesis Genes in Aspergillus nidulans
Source: mBio. 2021 Jun 22;12(3):e00768-21. doi: 10.1128/mBio.00768-21 (PMC8262921; doi:10.1128/mBio.00768-21)
Supplement: TABLE S1 [file mbio.00768-21-st001.pdf]

Table S1. Leucine biosynthesis genes genomic PCR primer sets.

Target	Primer Name[a]	Sequence (5' → 3')
batA	An4323FGSC5'F	CTCAGACGAGCGAGATTAC
	An4323FGSC3'R	CTACGGATCCCTACCTAGAA
	ANID_04323.1 5'F	GTAACGCCAGGGTTTTCCCAGTCACGACGCTCAGACGAGCGAGATTAC
	ANID_04323.1 3'R	GCGGATAACAATTTCACACAGGAAACAGCCTACGGATCCCTACCTAGAA
batB	An5957FGSC5'F	CTAGTGCAGCAAGAGATCC
	An5957FGSC5'F	AAGGACGACTTGAGGACTAC
	ANID_05957.1 5'F	GTAACGCCAGGGTTTTCCCAGTCACGACGCTAGTGCAGCAAGAGATCC
	ANID_05957.1 3'R	GCGGATAACAATTTCACACAGGAAACAGCAAGGACGACTTGAGGACTAC
batC	AN7878FGSC5'F	GATGAGTTCCCATAGTGAGC
	AN7878FGSC3'R	GGGGACTCATTAGTGAGAAG
	ANID_07878.1 5'F	GTAACGCCAGGGTTTTCCCAGTCACGACGGATGAGTTCCCATAGTGAGC
	ANID_07878.1 3'R	GCGGATAACAATTTCACACAGGAAACAGCGGGGACTCATTAGTGAGAAG
batD	ANID_07876.1 5'F	GTAACGCCAGGGTTTTCCCAGTCACGACGGCTAGATCTGACTCTTTGGC
	ANID_07876.1 3'R	GCGGATAACAATTTCACACAGGAAACAGCGTGCATATCTACATGGGTGG
batE	AN0385_5'F	GATTCCTGAGTAGGGATACG
	AN0385_3'R	GAGTCTGAATCTGCCTCTG
	ANID_00385.1 5'F	GTAACGCCAGGGTTTTCCCAGTCACGACGGATTCCTGAGTAGGGATACG
	ANID_00385.1 3'R	GCGGATAACAATTTCACACAGGAAACAGCGAGTCTGAATCTGCCTCTG
batF	An8511FGSC5'F	GCTCATGATACTAGCCCTCT
	An8511FGSC3'R	GGCTAGTACTGCTTTGATCC
	ANID_08511.1 5'F	GTAACGCCAGGGTTTTCCCAGTCACGACGGCTCATGATACTAGCCCTCT
	ANID_08511.1 3'R	GCGGATAACAATTTCACACAGGAAACAGCGGCTAGTACTGCTTTGATCC
leuD	AN0921FGSC5'F	ATCATCCTCTCTACTCCAGC
	AN0921FGSC3'R	GTATGTGGGTAGCATACTCG
	ANID_00921.1 5'F	GTAACGCCAGGGTTTTCCCAGTCACGACGTGTACGAGACTTCTACTGCC
	ANID_00921.1 3'R	GTAACGCCAGGGTTTTCCCAGTCACGACGTGTACGAGACTTCTACTGCC
leuE	AN2793FGSC5'F	TAGTTGGTACGTAGGTGGC
	AN2793FGSC3'R	GTGAAAGGAGAGATGAGGAG
	ANID_02793.1 5'F	GTAACGCCAGGGTTTTCCCAGTCACGACGTAGTTGGTACGTAGGTGGC
	ANID_02793.1 3'R	GCGGATAACAATTTCACACAGGAAACAGCGTGAAAGGAGAGATGAGGAG

[a] Underlined primers were used to generate the deletion constructs.
