# Supplementary material for: Duplication and Functional Divergence of Branched-Chain Amino Acid Biosynthesis Genes in Aspergillus nidulans
Source: mBio. 2021 Jun 22;12(3):e00768-21. doi: 10.1128/mBio.00768-21 (PMC8262921; doi:10.1128/mBio.00768-21)
Supplement: TABLE S2 [file mbio.00768-21-st002.pdf]

Table S2. Leucine biosynthesis gene RT-qPCR primers

Target	Primer Name	Sequence (5' → 3')	Efficiency[a] (54.0ºC)
benA	benA_RT_F	CCTGCTCCGCTSTCTTCC	87.8 %
	benA_RT_R	GACTGTTCTTGCTCTGGAT	
leuB	leuB_RT_F	GCTGTTTGACCTTTTCTTC	135.3 %
	leuB_RT_R	AAAGTAAGGGAGAGACAGT	
leuC	leuC_RT_F	TCAAAGATCCCTCTAAGAAATACA	155.4 %
	leuC_RT_R	TTGTTCGCCATCCATAGG	
luA	luA_RT_F	TCTTGATCTATATCGACAGACA	136.2 %
	luA_RT_R	TGACGAGGTAGGGATGTT	
leuD	leuD_RT_F	ATTGGTGGCCCAGAATGG	125.15 %
	leuD_RT_R	AATTGCATGGTCGCAAGTTG	
leuE	leuE_RT_F	AAATCTGAGACCGTGTTC	107.3 %
	leuE_RT_R	ATTCGTCCATTGCATAATC	
batA	batA_RT_F	GCTTCAGTGCTACTCGTG	137.1 %
	batA_RT_R	TGAACATGTGATCGGTAAAG	
batB	batB_RT_F	CGAAGTTGTTGAGAAGAC	133.3 %
	batB_RT_R	TGATGAAGAATGCAGTCC	
batC	batC_RT_F	TCTTTCACTGACCACATC	90.23 %
	batC_RT_R	TTATCGTAGGGAGTGATTTG	
batD	batD_RT_F	GGGATGAAAGCCTATCGT	ND[b]
	batD_RT_R	TAGAAACAGCATCACAAGAAC	
batE	batE_RT_F	CTCAATCATTCCCTCCTCCTC	98.0 %
	batE_RT_R	CATGGCCGTTAACTTCGC	
batF	batF_RT_F	AAGTTCGGTAAATGGTCTG	ND[b]
	batF_RT_R	CTCCTGTCCGTAGTTGAA	

[a] Efficiency was calculated from a serial dilution with 3-5 concentrations.

[b] Not determined; expression was undetectable or too low to perform serial dilution analysis.
